# Ultra-selective molecular-sieving gas separation membranes enabled by multi-covalent-crosslinking of microporous polymer blends

**DOI:** 10.1038/s41467-021-26379-5

**Published:** 2021-10-22

**Authors:** Xiuling Chen, Yanfang Fan, Lei Wu, Linzhou Zhang, Dong Guan, Canghai Ma, Nanwen Li

**Affiliations:** 1 State Key Laboratory of Coal Conversion, Institute of Coal Chemistry, Chinese Academy of Sciences, Taiyuan, 030001 China; 2 Hubei Key Laboratory of Radiation Chemistry and Functional Materials, Hubei University of Science and Technology, Xianning, 437100 China; 3 State Key Laboratory of Heavy Oil Processing, College of Chemical Engineering and Environment, China University of Petroleum-Beijing, Beijing, 102249 China; 4 State Key Laboratory of Fine Chemicals, Research and Development Center of Membrane Science and Technology, School of Chemical Engineering, Dalian University of Technology, Dalian, Liaoning 116024 China

**Keywords:** Energy supply and demand, Health occupations

## Abstract

High-performance membranes exceeding the conventional permeability-selectivity upper bound are attractive for advanced gas separations. In the context microporous polymers have gained increasing attention owing to their exceptional permeability, which, however, demonstrate a moderate selectivity unfavorable for separating similarly sized gas mixtures. Here we report an approach to designing polymeric molecular sieve membranes via multi-covalent-crosslinking of blended bromomethyl polymer of intrinsic microporosity and Tröger’s base, enabling simultaneously high permeability and selectivity. Ultra-selective gas separation is achieved via adjusting reaction temperature, reaction time and the oxygen concentration with occurrences of polymer chain scission, rearrangement and thermal oxidative crosslinking reaction. Upon a thermal treatment at 300 °C for 5 h, membranes exhibit an O_2_/N_2_, CO_2_/CH_4_ and H_2_/CH_4_ selectivity as high as 11.1, 154.5 and 813.6, respectively, transcending the state-of-art upper bounds. The design strategy represents a generalizable approach to creating molecular-sieving polymer membranes with enormous potentials for high-performance separation processes.

## Abstract (web summary)

Microporous polymers become increasingly attractive as materials for the fabrication of permeable and selective gas separation membranes but separation performance is often limited by broad pore size distribution. Here, the authors design a porous polymer membrane via multi-crosslinking of miscible blends of microporous polymers enabling simultaneous high permeability and selectivity.

## Introduction

The energy associated with the separation and purification of industrial gases, fine chemicals, and water, currently accounts for 10–15% of national total energy consumption^1^. Such number is projected to triple by 2050^2,3^. Global energy scarcity, along with climate change and rapid growth of population, stimulates the exploration of energy-efficient technologies for gas separations, water purification, and energy generation^4–6^. Owing to advantages of low-energy consumption, small footprint, and easy operation^7–9^, membrane-based separation technologies hold great promise to address the need of energy-efficient separation processes. Currently, gas separations based on membranes mostly rely on synthetic polymeric materials with customizable gas transport properties. Ultra-permeable and selective polymeric membranes have become a critical factor to achieve the most desirable recovery and purity of gas products in the practical industrial applications.

To enhance the gas separation performance of polymeric membranes, various material synthesis and design strategies have been proposed and studied extensively, including adjustment of the rigidity of polymers through grafting bulky groups^10–13^, construction of hybrid membranes via integrating molecular sieve fillers into polymers^14–16^, and modification of polymer structures at microscopic levels by external stimulus (e.g., heat, light, and oxygen)^17–19^. For example, rigid polymers with high free volume, such as polymers of intrinsic microporosity (PIMs)^20^, Tröger’s base polymers (TB), and bulky groups containing polyimides^21^, have demonstrated several orders of magnitude higher gas permeability than commercial polymer membranes, such as Matrimid^22^. The disrupted polymer chain packing in these rigid polymers results in the formation of microcavities in the solid state, allowing fast diffusion of gases without significantly losing selectivity. Additionally, the polymer matrices incorporated with inorganic or organic molecular sieves, such as covalent organic frameworks, metal organic frameworks, and graphene oxides, provide alternatives to obtain membranes with enhanced gas separation performance^23,24^. Subsequently, crosslinking or rearrangement of aforementioned polymer systems induced by chemical, light, or heat treatment offer further approaches to tailor microscopic structures of polymers^17–19^. Although all of these approaches demonstrate potentials to prepare membranes with promising gas separation properties, microstructural engineering of polymers to narrow the pore size distribution and enhance molecular sieving properties has remained a crucial challenge for high-performance gas separation membranes.

Herein, we developed a methodology for fabricating multi-covalent crosslinked microporous membranes, exhibiting advantages of mild membrane processing temperatures and excellent molecular sieving properties. Unlike the above-mentioned studies on crosslinked polymers through external stimulus, which mostly involved the intra-molecular crosslinking reaction and could hardly tune the polymer microstructure finely, we established the strategy of oxygen-induced chain scission, polymer segment rearrangement alongside in situ intra/inter polymer chain crosslinking to construct hyper-crosslinked networks in a microporous polymer system under a moderate temperature.

**Fig. 1:**
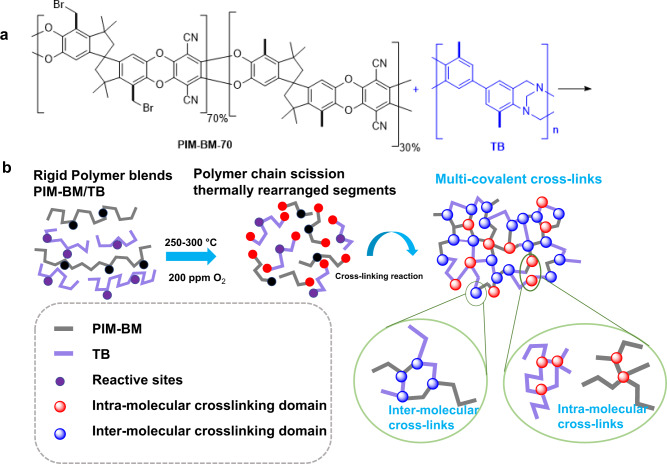
Schematic of crosslinking reactions between PIM-BM and TB. **a** Chemical structure of PIM-BM and TB. **b** Proposed crosslinking mechanisms between PIM-BM and TB.

Our strategy is specially designed for the polymer blends of bromomethylated PIMs (PIM-BM) and TB, which are judiciously selected as a prototype of microporous polymer system to simultaneously provide inter- and intra-molecular crosslinking reaction sites (Fig. 1). The nucleophilic coupling reactions between reactive sites of CH_2_Br groups of PIM-BM and tertiary amino groups of TB in PIM-BM/TB blends play a critical role of creating a pre-crosslinking network prior to oxidative crosslinking reaction. This pre-crosslinking reaction is expected to affect polymer chain mobility significantly, and polymer chain scission occurs at above 250 °C upon exposure to ppm-level O_2_, enhancing molecular sieving properties of membranes. It is believed that multiple crosslinking reactions occurring progressively at different temperatures contribute to the formation of molecule sieving structures in crosslinked PIM-BM/TB membranes (denoted as XPIM-BM/TB). The resultant XPIM-BM/TB membranes exhibit ultrahigh selectivities with respect to technically important gas pairs such as H_2_/CH_4_, CO_2_/CH_4_, and O_2_/N_2_. Compared to crosslinked PIM-1^18^ and oxidatively crosslinked PIM-BM, TB (Supplementary Fig. 21), or PIM-1^17^ as reported in literatures previously, our membranes demonstrate the highest CO_2_/CH_4_ selectivity up to 154.5 with a CO_2_ gas permeability of 68 Barrer. This fact indicates that the intra-molecular crosslinking reaction occurring in either pure TB or PIM-1 alone does not create desired polymer membrane structure to achieve the ultrahigh gas selectivities. Moreover, the membranes display a H_2_/CH_4_ selectivity as high as 813.6 with a H_2_ permeability of 358 Barrer. In comparison with intra-molecularly crosslinked PIM-BM, XPIM-BM/TB membranes perform far beyond the current permeability-selectivity upper bounds for multiple gas pairs (e.g., CO_2_/CH_4_, H_2_/CH_4_, H_2_/N_2_, O_2_/N_2_). We attribute such high separation performance to finely tuned pore size distribution in crosslinked membranes through integrated multi-covalent crosslinking reactions including self-crosslinking within PIM-BM and inter/intra-molecular crosslinking of polymer blends (PIM-BM and TB). In any case, the membranes demonstrate appealing gas separation performance enabling ultra-selective separation of industrially relevant gas pairs as discussed in this work.

## Results

### Materials synthesis and characterizations

The N atom in the TB moieties can interact with –CN groups in PIMs, considerably improving the miscibility of PIMs and TB polymer. Therefore, in this work, the bromomethylated PIMs were prepared through converting methylated PIM-1 to PIMs with bromomethyl groups by using *N*-bromosuccinimideas bromomethylating agent (represented by PIM-BM-*x*, *x* refers to the degree of bromomethylation and *x* = 70, Supplementary Figs. 1–3)^25^. Then the blends of PIM-BM-70 with rigid TB polymers (Supplementary Figs. 4 and 5) with excellent mechanical properties (PIM-BM/TB) (Supplementary Fig. 19a and Supplementary Table 3) were selected as the framework to develop crosslinked microporous membranes.

**Fig. 2:**
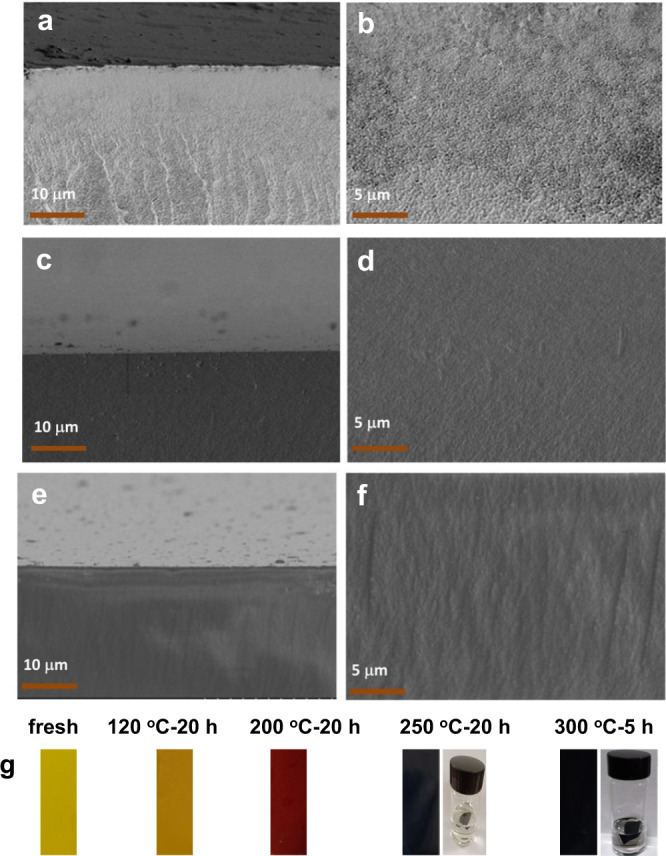
SEM images and photos of polymer membranes after thermal crosslinking at varied temperatures. **a**, **c**, **e** Cross-sectional images of PIM-BM/TB, PIM-BM/TB-250 °C-10 h and PIM-BM/TB-300 °C-5 h. **b**, **d**, **f** Surface images of PIM-BM/TB, PIM-BM/TB-250 °C-10 h and PIM-BM/TB-300 °C-5 h. **g** Photos of PIM-BM/TB treated at different temperatures.

Physically blended PIM-BM-70 and TB polymers exhibited excellent miscibility and were readily fabricated into transparent membranes by dissolution in chloroform and casting on a glass plate. The resultant PIM-BM/TB membranes were crosslinked by thermally processing in a temperature window of 120–300 °C over varied periods in nitrogen with ppm-level oxygen. The cross-sectional morphology of membranes was characterized by scanning electron microscopy (SEM). As the SEM images show in Fig. 2a–f, all membranes regardless of thermal crosslinking temperatures display smooth and macrovoid-free surfaces. With the increase of thermal processing temperature from 120 to 300 °C, the color of membranes changes from original yellow to brown yellow, and then to black as observed in Fig. 2g. PIM-BM/TB treated at 200 °C over 20 h is partially insoluble in common organic solvents such as chloroform (Supplementary Fig. 10), which can easily dissolve the pristine PIM-BM/TB. Upon treatment at high temperatures between 250 and 300 °C, PIM-BM/TB membranes become completely insoluble in chloroform as depicted in Fig. 2g.

We proposed three types of chemical crosslinking mechanisms likely occurring during the heating process, i.e., reactions of tertiary amine with bromomethyl groups with the formation of quaternary ammonium salts, alkylation reactions, and oxidative crosslinking reactions within PIM-BM/TB membranes as schematically shown in Fig. 1 and Supplementary Figs. 7–9.

**Fig. 3:**
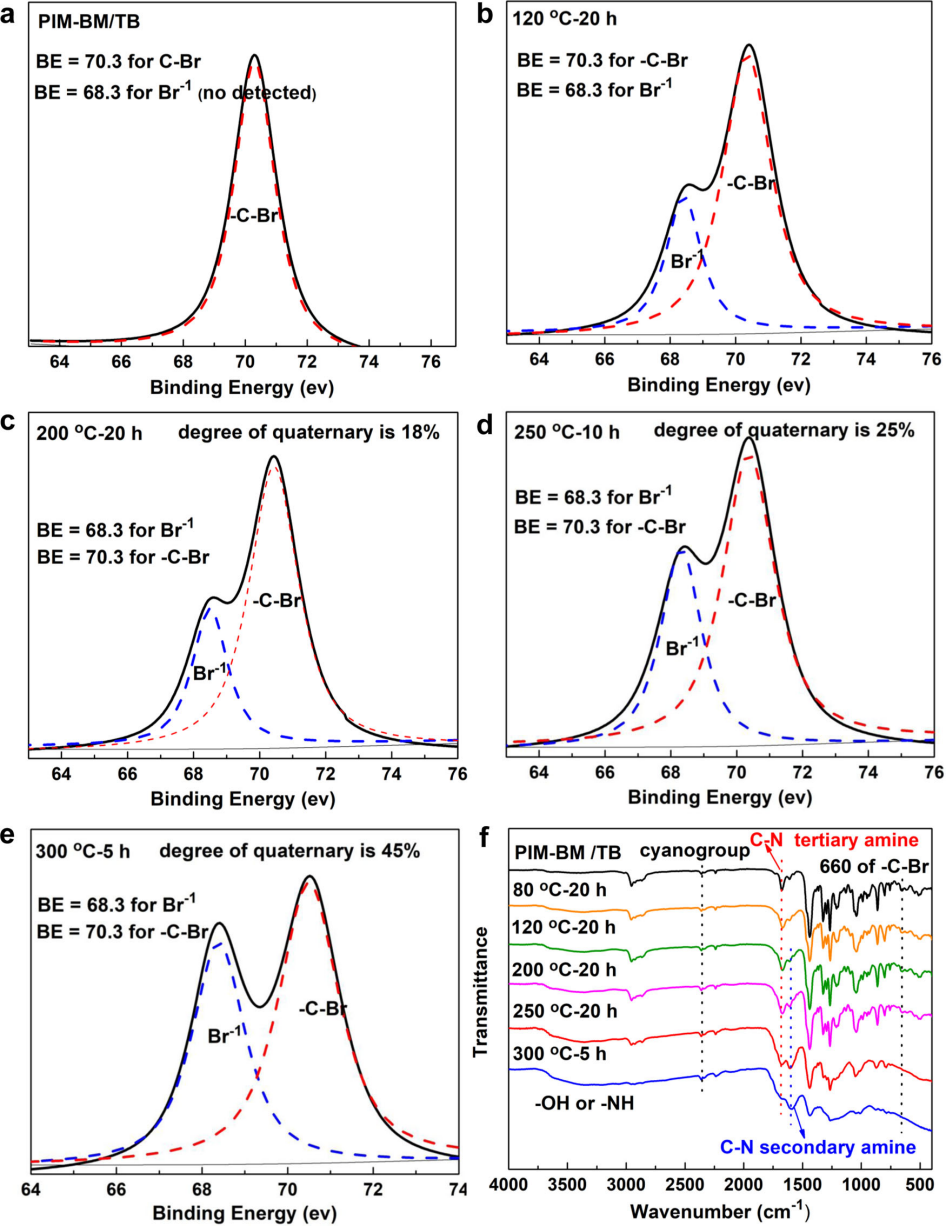
Structural characterizations of fresh and thermally crosslinked PIM-BM/TB. **a** XPS spectrum of Br 3*d* of fresh PIM-BM/TB. **b** XPS spectrum of Br 3*d* of PIM-BM/TB-120 °C-20 h. **c** XPS spectrum of Br 3*d* of PIM-BM/TB-200 °C-20 h. **d** XPS spectrum of Br 3*d* of PIM-BM/TB-250 °C-10 h. **e** XPS spectrum of Br 3*d* of XPIM-BM/TB-300 °C-5 h. **f** FTIR spectra of fresh and thermally crosslinked PIM-BM/TB.

In the first scenario, we found the critical role of reactive sites of CH_2_Br groups of PIM-BM and tertiary amino groups of TB in PIM-BM/TB blends. The –CH_2_Br groups in repeat units of PIM-BM react with tertiary amino groups in TB polymers. X-ray photoelectron spectrometer (XPS) results (Fig. 3a–e) confirm that covalent C–Br bonds in fresh PIM-BM/TB membranes are gradually transformed into Br-containing salts via nucleophilic coupling reactions of tertiary amine with bromomethyl groups during the initial thermal treatment. This reaction occurs at a temperature of 120–300 °C. The extent of nucleophilic coupling reactions between C–Br bonds and tertiary amine quantified by XPS results is found to increase from 12% to 40% with the reaction temperature increasing from 120 to 300 °C (Supplementary Table 2). Thus, the primary reaction mechanism proposed that partial tertiary amino groups in TB polymers and bromomethyl groups in PIM-BM (bromomethylated PIMs) are converted to quaternary ammonium salts [N^+^(R)_3_]CH_2_RBr^−^ through coupling reactions (Supplementary Fig. 7). Based on such reaction mechanisms, the crosslinking degrees of XPIM-BM/TB-250 °C-10 h and XPIM-BM/TB-300 °C-5 h membranes are estimated from XPS results to be 25% and 40%, respectively.

With respect to the second reaction mechanism, the membranes were subjected to inert gas with ppm-level oxygen in the temperature range of 250–300 °C. In this case, we proposed a possible thermal crosslinking of C–Br bonds and benzene rings via alkylation reaction route, generating HBr as a gaseous by-product, besides the aforementioned coupling reaction (Supplementary Figs. 8 and 9). As evidenced by the ion current corresponding to HBr from TG/mass spectrometry (Supplementary Fig. 11a), HBr is released from 230 to 300 °C for pristine PIM-BM/TB, corroborating the crosslinking mechanism through alkylation reaction. In contrast, the amount of HBr released during alkylation reaction decreases significantly for the case of crosslinked XPIM-BM/TB-250 °C-10 h, since most of CH_2_Br groups have participated in the crosslinking reaction with benzene rings. TG-MS did not detect HBr signal for XPIM-BM/TB-300 °C-5 h (the trace amount of HBr was difficult to detect), suggesting consumption of the majority of C–Br groups as reactions proceed within the polymer blends. The depletion of C–Br groups was also verified by XPS results, where Br 3*d* core signal ratio of C–Br (70.3 eV) to Br^−^ (68.3 eV) in XPS spectra significantly decreased for the membrane thermally treated at 300 °C for 5 h (Fig. 3e). Under the alkylation reaction, the crosslinking degrees of XPIM-BM/TB-250 °C-10 h and XPIM-BM/TB-300 °C-5 h membranes estimated from XPS results are 22% and 45%, respectively. Moreover, as depicted by Fourier transform infrared spectra (FTIR) of the membranes (Fig. 3f), the characteristic peak of C–Br near 660 cm^−1^ for XPIM-BM/TB membranes treated at temperatures ≥250 °C becomes less intense compared with the original PIM-BM/TB, further supporting the consumption of C–Br groups during the crosslinking reaction.

In the third stage, we proposed an oxidative crosslinking mechanism that PIM-BM/TB membranes experienced a thermally oxidative reaction during the heating treatment under a temperature of 250–300 °C in the presence of ppm-level oxygen. Such phenomena was also observed in the temperature range of 300–450 °C for pure PIM-1^17^. The oxygen plays a key role in partially decomposing polymer chains into polymer fragments. The scission of spiro linkage of PIM-BM and methylene groups linked to N atoms of TB is likely the dominant decomposition step. Partial backbones are oxidized, leading to the formation of COOH groups^17^. Afterwards, fragmented polymer chains are hypothetically thermally rearranged to an energy-favorable state. Simultaneously, the chemical reaction takes place among reactive groups including oxidation-induced free radicals, CH_2_Br, and N-containing groups in polymer chains, leading to extensive covalent crosslinking (Fig. 1). The degree of oxidative polymer chain scissor is tunable via changing the oxygen concentration in the purge gas as will be discussed later.

Note that the IR spectra of the 300 °C crosslinked PIM-BM/TB membrane demonstrate distinct peaks of the functional organic groups of samples, indicating a polymer nature of the membrane (Fig. 3f). To further clarify the state of membranes, an elemental analysis was conducted using treating temperatures up to 550 °C, a temperature widely used in pyrolyzing membranes (Supplementary Table 15)^33^. The carbon content and C/O ratio of the 300 °C-treated membranes are quite close to the values of untreated membranes but significantly lower than that of 550 °C-carbonized samples. A polymer state of PIM-1 membranes crosslinked at a temperature as high as 385 °C was also reported in literatures^17^. Based on those evidence, it is believed that the crosslinked membranes remain in a polymer state instead of being carbonized.

As discussed above, despite the different trends observed in PIM-BM/TB membrane characterizations under various temperatures, the three possible crosslinking mechanisms are inherently coupled, all of which can contribute to the crosslinked network formed within the membranes. Decoupling the crosslinking reaction mechanisms in PIM-BM/TB membranes merits further study to clarify the effects of each individual reaction on membrane structures and properties.

### Membrane characterizations

**Fig. 4:**
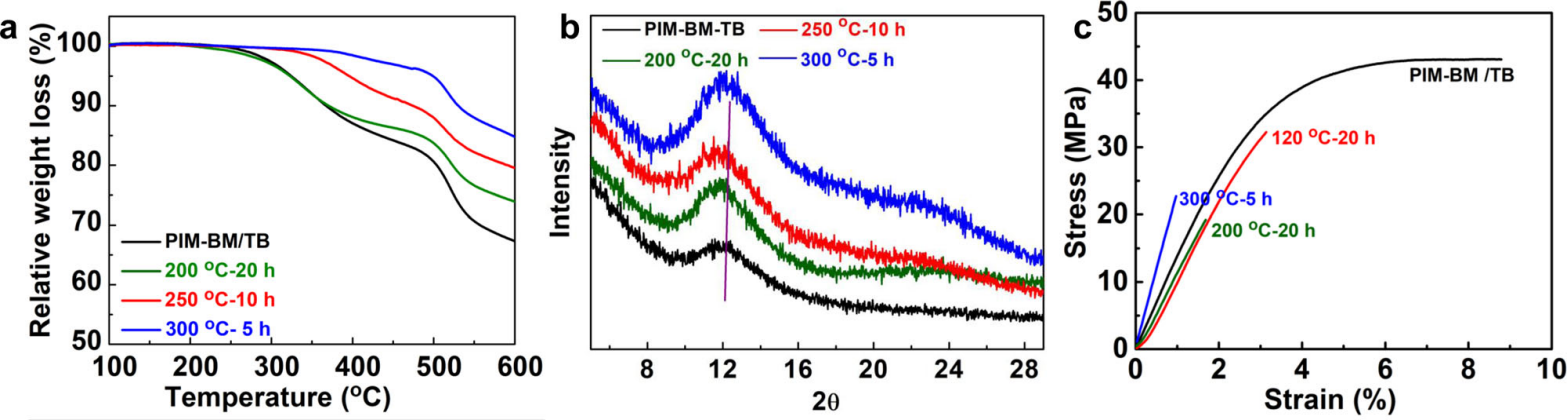
Properties of crosslinked microporous membranes. **a** Thermal stability of PIM-BM/TB and crosslinked PIM-BM/TB. **b** XRD profiles. **c** Mechanical properties of polymer membranes.

The physiochemical properties of membranes were further characterized using a wide range of techniques. As TGA profiles show, the dependence of weight loss on crosslinking temperatures demonstrates that crosslinked membranes have improved thermal stability over uncrosslinked PIM-BM/TB blends (Fig. 4a and Supplementary Fig. 11a). In fact, the XPIM-BM/TB-300 °C-5 h membranes start to decompose at ~400 °C, significantly higher than the case of untreated PIM-BM/TB membranes or treated at 200 °C with a decomposition temperature of ~250 °C. X-ray diffraction (XRD) spectra of PIM-BM/TB before and after thermal crosslinking reveal that all polymers are amorphous (Fig. 4b). Referring to XRD profiles of pristine membranes, the peak at the angle of 11.9° (*d*-space values of 7.43 Å) is attributed to loosely packed polymer chains^26,27^. With increasing crosslinking extent, the broad peaks at 11.9° slightly shift to a higher angle (i.e., a smaller *d*-space value), implying that crosslinking reactions tighten the chain spacing and potentially enhance molecular sieving properties of membranes. A molecular modeling comparison of PIM-BM/TB and XPIM-BM/TB shows reduced fractional free volume with increasing crosslinking degree (Supplementary Table 1), consistent with PALS results.

A typical plot of stress–strain curve of membranes (Fig. 4c) depicts a reduction in elongation at break and tensile strength with increasing crosslinking degree, suggesting that the XPIM-BM/TB films become less ductile compared with untreated PIM-BM/TB. For example, pristine membranes have an ultimate yield stress of 43 MPa at 8.8% strain, whereas the membranes treated at 200 °C for 20 h have a tensile stress of 19 MPa at 1.9% strain at break. When further increasing the temperature to 300 °C, despite a decrease of elongation at break, the membranes maintain a high mechanical stress of 22 MPa (Supplementary Table 3). The mechanical-strength tests demonstrate potentials of preparing robust membranes using our approach.

### Pore structure characterization

**Fig. 5:**
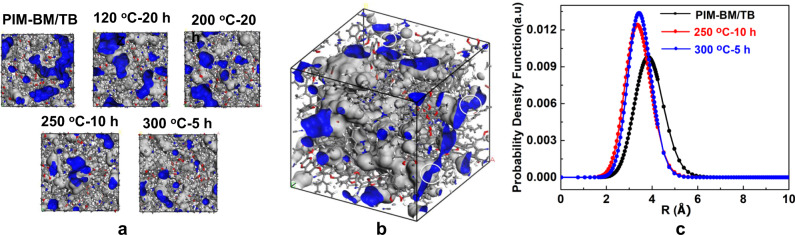
Characterization of PIM-BM/TB and XPIM-BM/TB membranes physical structures. **a** Representative chain conformations in crosslinked PIM-BM/TB from computer modeling results. **b** 3-D view of crosslinked PIM-BM/TB modeling structure in an amorphous cell (300 °C-5 h) (cell size: 30 × 30 × 30 A; density: ~1.223 g/cm^3^; Gray–Van der Waals surface; dark gray–Connolly surface with pore radius of 1.45 A). **c** Pore size distribution from PALS.

As evidenced by the above XRD testing, the formation of covalent crosslinking bonds tends to tighten the polymer chains, leading to a reduced *d*-spacing in polymer matrix. To gain further insights of microstructures, the pore size distribution of membranes was characterized using combined simulated and experimental approaches based on the MELT program and PALS results. As visualized by molecular simulation in Fig. 5a, the rigid polymer chains in PIM-BM/TB are disorderly packed, leading to the formation of irregularly shaped free volume. Molecular modeling comparison of pristine PIM-BM/TB with crosslinked PIM-BM/TB shows the occurrence of efficient packing after crosslinking (Fig. 5a, b). This crosslinking-induced tightening effect is consistent with the fractional free volume (FFV) simulation (Supplementary Table 1). For instance, using H_2_ as the structure probe, the calculated fractional free volume was reduced from 0.228 to 0.187 for the uncrosslinked PIM-BM/TB and crosslinked XPIM-BM/TB-300 °C-5 membranes, respectively.

Figure 5c reveals the free volume distributions generated from MELT program based on PALS results. The average free volume radii shift to a lower value upon crosslinking. As a result, crosslinked membranes exhibit a smaller fractional free volume than untreated samples, as described in Table S1. Moreover, a thermal treatment at 250 and 300 °C results in a much narrow pore size distribution over untreated membranes, indicating the more restricted chain motion and smaller cavities in the crosslinked format of membranes^28^. Indeed, crosslinking not only tightens the inner pores of membranes but also tailors the width of ultramicropores connecting neighboring cavities, allowing selective diffusion of smaller gas molecules such as H_2_ and CO_2_, while excluding larger gas molecules like N_2_ and CH_4_, which will be discussed in the section below.

### Gas transport properties

**Fig. 6:**
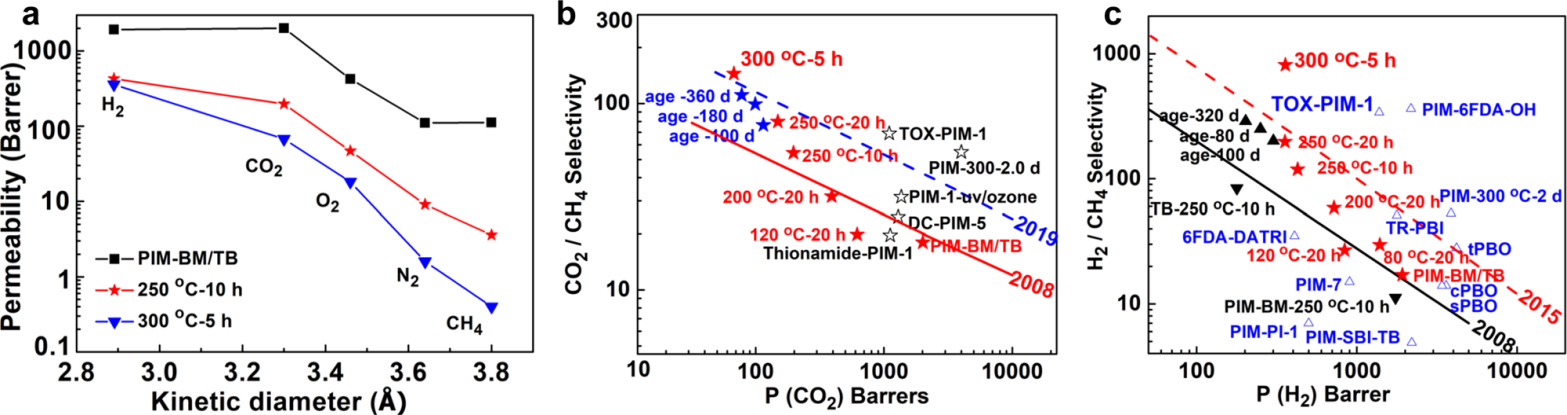
Gas transport properties. **a** Gas permeabilities as a function of kinetic diameters. **b** CO_2_/CH_4_ separation with Robeson upper bound. **c** H_2_/CH_4_ separation with upper bounds.

To explore the gas transport properties in the membranes, single gas permeation was first conducted on pristine polymer membranes and thermally crosslinked membranes using gas molecules including H_2_ (2.89 Å), CO_2_ (3.3 Å), O_2_ (3.46 Å) N_2_ (3.64 Å), and CH_4_ (3.8 Å) at 35 °C under a feed pressure of 50 psia. Gas permeabilities and ideal selectivities of the membranes are described in Table 1. The pristine PIM-BM/TB membranes exhibit high permeability with moderate gas selectivities, consistent with reported PIMs family membranes. As expected, the crosslinking reaction results in a drastic decrease in gas permeability for XPIM-BM/TB due to pore structure shrinkage as discussed above. Particularly, the gas permeability of large gas molecules (CH_4_, N_2_) decreases much more significantly over smaller gas molecules like O_2_, H_2_, CO_2_ as shown in Fig. 6a. Nevertheless, the gas selectivity increases remarkably upon thermal crosslinking. Moreover, the sequence of gas permeability follows the order of H_2_ > CO_2_ > O_2_ > N_2_ > CH_4_ in XPIM-BM/TB regardless of crosslinking temperatures. Such trend is in accordance with the order of gas kinetic diameters, suggesting the molecular sieving property of these membranes. Indeed, the crosslinked membranes with efficiently packed chains display substantially enhanced molecular sieving capability. For instance, the H_2_/CH_4_ selectivity of representative XPIM-TM/TB membranes prepared upon heating at 250 °C for 10 h increases from 17.2 to 118.6 and CO_2_/CH_4_ selectivity increases from 17.9 to 54.7.

**Table 1:** Gas permeabilities and selectivities of uncrosslinked PIM-BM/TB and crosslinked XPIM-BM/TB.

Polymer	*P* (Barrer)^a^					Selectivity^b^			
	H_2_	CO_2_	O_2_	N_2_	CH_4_	H_2_/CH_4_	CO_2_/CH_4_	O_2_/N_2_	CO_2_/N_2_
PIM-BM/TB	1925	2007	423	110	112	17.2	17.9	3.8	18.2
80 °C-20 h	1392	987	216	44	47	29.6	21.0	4.9	22.4
120 °C-20 h	839	618	150	33	31	27.1	19.9	4.5	18.7
200 °C-20 h	721	391	88	18	12	60.1	32.6	4.9	21.7
250 °C-5 h	582	431	104	25	16	36.4	26.9	4.2	17.2
250 °C-10 h	427	197	47	9	3.6	118.6	54.7	5.2	21.8
250 °C-20 h	356	149	33	4.4	1.8	197.7	82.7	7.5	33.9
300 °C-5 h	358	68	18	1.6	0.44	813.6	154.5	11.1	42.5

^a^Permeability coefficients measured at 35 °C and 50 psi feed pressure. 1 Barrer = 10^−10^ [cm^3^ (STP) cm]/(cm^2^ s cmHg).

^b^Ideal selectivity ɑ = (*P*_*a*_)/(*P*_*b*_).

The CO_2_/CH_4_ and H_2_/CH_4_ separation data are plotted with the current upper bounds and compared with literatures. Figure 6b, c shows the remarkably high gas selectivity over other reported PIM-based membranes. In comparison to industrially used polymeric gas separation membranes (e.g., polysulfone, PSF), the XPIM-BM/TB membranes demonstrate apparently higher selectivities while maintaining orders-of-magnitude higher permeability, well exceeding the present upper bounds^29–31^. Notably, the H_2_/CH_4_ selectivity of XPIM-BM/TB membranes treated at a temperature of 300 °C for 5 h is as high as 813.6, increased by 47-fold over uncrosslinked PIM-BM/TB, which is among the highest values reported for comparable polymeric membranes. Owing to rigid structures with closely packed chains in these membranes, a significantly high H_2_/CH_4_ permselectivity is achieved. The relatively smaller reduction of H_2_ than the case of CH_4_ after thermal treatment indicates the formation of an ideally narrow pore structure of XPIM-BM/TB, preferentially permeating H_2_ over CH_4_; thereby an ultrahigh H_2_/CH_4_ selectivity is obtained.

Other notable gas pairs including O_2_/N_2_ and H_2_/N_2_ are described in Supplementary Fig. 18. Similar to H_2_/CH_4_, the PIM-BM/TB membranes crosslinked under 300 °C-5 h display considerably high O_2_/N_2_ and H_2_/N_2_ selectivities and substantially exceed the gas separation limits of conventional polymeric membranes. The separation process of O_2_/N_2_ is much more difficult as compared to H_2_/CH_4_ and CO_2_/CH_4_ since O_2_ and N_2_ have similar kinetic diameters with only Ångstrom-level differences. Nonetheless, the crosslinked membranes exhibit great promise for O_2_/N_2_ separation as the separation performance is located well above the Robeson upper bound. With increasing reaction temperature, the O_2_/N_2_ selectivity remarkably increases from 3.8 to 11.1 with a reduction of O_2_ permeability from 423 to 18 Barrer. An impressive O_2_/N_2_ selectivity over 11 is achieved in this work, comparable with the most-selective membrane materials for O_2_/N_2_ separation summarized by Robeson^32^.

In any case, the ultra-selective XPIM-BM/TB membranes developed in this work are among the best-performing membranes reported in the literature for separating gas pairs including CO_2_/CH_4_, H_2_/CH_4_, H_2_/N_2_, and O_2_/N_2_ (Fig. 6 and Supplementary Figs. 17 and 18).

### Tunable gas transport properties

**Fig. 7:**
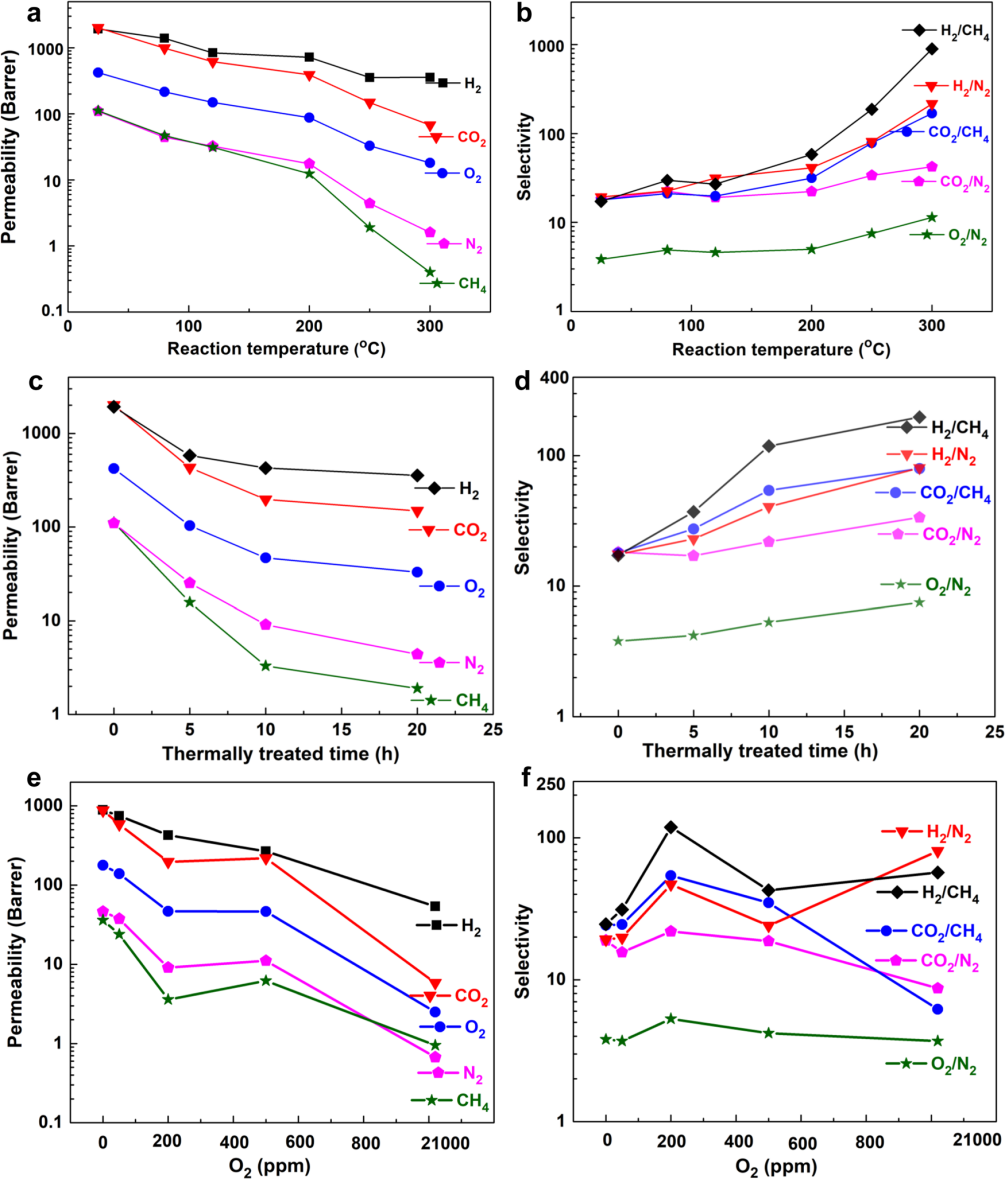
Gas transport properties of membranes prepared under various conditions. **a** Gas permeability and **b** gas selectivity as a function of reaction temperature. All samples were thermally treated at set-point temperature for 10–20 h under 200 ppm of O_2_, except that the sample at 300 °C was annealed for 5 h. **c** Gas permeability and **d** gas selectivity as a function of reaction time treated at 250 °C of crosslinked membranes. **e** Gas permeability and **f** gas selectivity as a function of oxygen concentrations for membranes treated at 250 °C of 10 h.

One of the important advantages of PIM-BM/TB polymer membranes is the versatility in tailoring the microporous structure and gas separation performance. The gas transport properties intimately correlated with pore structures of membranes can be precisely tuned by controlling crosslinking reaction temperature, reaction time, and the atmosphere. Figure 7 shows the effects of reaction temperature, reaction time, and oxygen concentration in the purging gas on gas permeability and selectivity in crosslinked PIM-BM/TB. Evidently, as the crosslinking temperature increases from 80 to 300 °C, the permeability gradually decreases while the selectivity increases significantly (Fig. 7a, b). In fact, the crosslinked membranes demonstrate a more substantial drop in gas permeability for larger molecules like CH_4_ and a less reduction for smaller molecules like H_2_ upon crosslinking (Table 1). Correspondingly, the H_2_/CH_4_ gas pair with the largest kinetic diameter difference is most sensitive to reaction temperatures, yielding membranes with the highest H_2_/CH_4_ selectivity among gas pairs explored in this study (i.e., H_2_/CH_4_, H_2_/N_2_, CO_2_/CH_4_, CO_2_/N_2_, and O_2_/N_2_).

In addition to the reaction temperature, the gas separation performance can be readily tailored by varying crosslinking time. For the membranes reacted with an identical reaction temperature of 250 °C, increasing crosslinking time from 5 to 20 h leads to the increase of gas selectivities along with a reduction in gas permeability. For example, the CO_2_ permeability decreases from 431 to 149 Barres and CO_2_/CH_4_ selectivity increases from 26.9 to 79.9 (Supplementary Table 6). We further investigated the effects of oxygen concentration on the gas separation performance of crosslinked membranes at a temperature of 250 °C with a reaction duration of 10 h as shown in Fig. 7e, f. For example, the CO_2_ gas permeability decreases when increasing oxygen concentration from inert gas to a level of 21,000 ppm. In particular, the gas selectivity of CO_2_/CH_4_ reaches the maximum of 54.7 at 200 ppm oxygen. The results in Fig. 7e, f clearly prove that the oxygen concentration plays a critical role during thermally oxidative crosslinking reaction. Based on the above discussions, adjusting the crosslinking temperature, time, and oxygen concentration can readily generate polymeric membranes with an attractive gas separation performance. To further tune the membrane performance, the blending ratio of PIM-BM/TB and degree of bromomethylation are other potential factors to improve the gas transport properties, which is currently being explored in our lab.

### Effects of feed pressures and ageing behavior

**Fig. 8:**
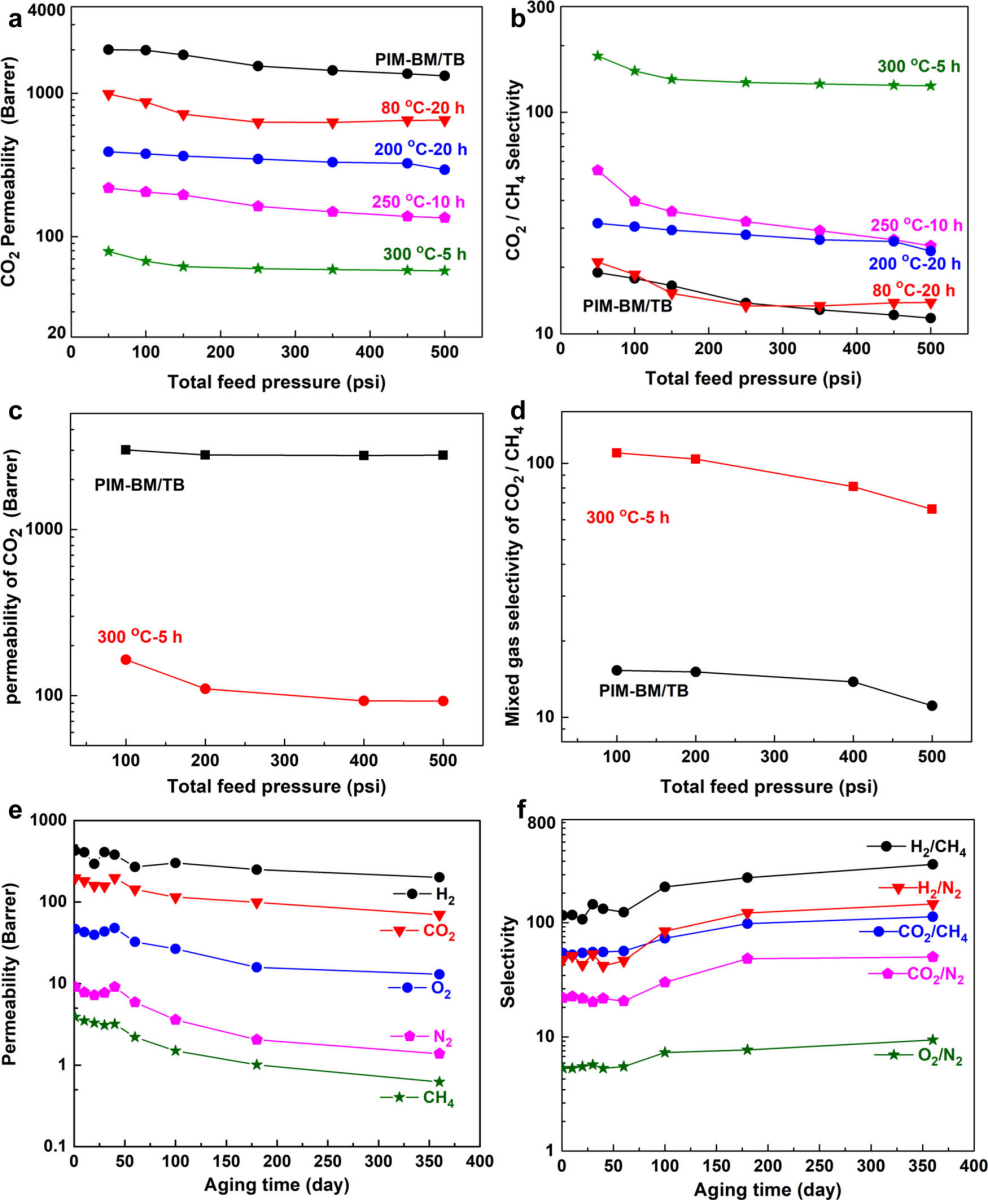
Effects of feed pressures and physical ageing on membranes. **a** Pure CO_2_ permeability versus feed pressure. **b** CO_2_/CH_4_ ideal selectivity versus feed pressure. **c** CO_2_ permeability versus feed pressure for separating equimolar CO_2_/CH_4_ gas mixtures. **d** CO_2_/CH_4_ selectivity versus feed pressure for separating equimolar CO_2_/CH_4_ gas mixtures. **e** Pure gas permeability versus aging time of XPIM-BM/TB- 250 °C-10 h. **f** Gas selectivity versus aging time of XPIM-BM/TB- 250 °C-10 h.

To explore the practical applicability of membranes under aggressive feed conditions, the membranes were subjected to high CO_2_ feed pressures. In the case of uncrosslinked PIM-BM/TB membranes, exposure of membranes to high-pressure pure CO_2_ leads to reduced gas permeability owing to the nonideality under high pressures (Fig. 8a), which is commonly observed in highly porous PIMs^34^. The CO_2_/CH_4_ selectivity decreases by increasing the pressure to 500 psia. On the other hand, the crosslinked membranes exhibit much less permeability decline over pristine membranes, indicating considerably stabilized structure in the membranes. Furthermore, PIM-BM/TB-300 °C-5 h were tested with equimolar CO_2_/CH_4_ gas mixture (Fig. 8c, d). Mixed gas permeation results show that the thermally treated PIM-BM/TB membranes have appealing mixed CO_2_/CH_4_ separation properties with CO_2_ permeability of 165 Barrer and CO_2_/CH_4_ selectivity of 110 tested at 100 psi. By further increasing the feed pressure up to 500 psi, both CO_2_ permeability and CO_2_/CH_4_ tend to decline; however, the crosslinked membranes maintain a CO_2_ permeability above 93 Barrer and CO_2_/CH_4_ selectivity above 66 at the maximal pressure of 500 psi, exhibiting desirable performance for operation under aggressive conditions.

To evaluate the long-term stability of membranes, physical ageing over a period of 360 days for XPIM-BM/TB treated at 250 °C for 10 h was investigated. Gas permeability of XPIM-BM/TB gradually decreases while gas selectivity increases during ageing. As shown in the Robeson plot (Fig. 6b, c), the aged XPIM-BM/TB membranes still demonstrate outstanding gas separation performance well above the 2008 Robeson upper bounds for both CO_2_/CH_4_ and H_2_/CH_4_.

## Discussion

In this work, we describe a method of designing microporous polymer blend membranes through multi-covalent-crosslinking of PIM-BM/TB. We propose three possible crosslinking mechanisms within PIM-BM/TB membranes that are dependent on crosslinking temperatures: (a) the formation of quaternary ammonium salts through reactions of tertiary amine with bromomethyl groups; (b) alkylation reactions at elevated temperatures; and (c) thermally oxidative crosslinking reactions after the polymer chain scission and rearrangements. The complex intra- and inter-crosslinking reactions simultaneously occur between PIM-BM and TB.

The crosslinked PIM-BM/TB molecular sieving membranes with tailorable porosity exhibit desired gas separation performance for industrially important gas pairs. The membranes display an ultrahigh H_2_/CH_4_ selectivity of 813.6 in crosslinked XPIM-BM/TB at 300 °C for 5 h while maintaining a H_2_ permeability of 358 Barrer. More importantly, the crosslinked membranes substantially exceed the present upper bound limits of conventional polymeric membranes for multiple gas pairs, including H_2_/CH_4_, CO_2_/CH_4_, H_2_/N_2_, and O_2_/N_2_. In the future, the physical and gas separation properties of microporous polymeric membranes developed in this work can be further tailored by controlling the degree of bromomethlyation and optimizing the blending ratio of PIM-BM to TB during polymer synthesis.

Overall, the strategy in this work provides a route of designing and fabricating promising molecular sieving membranes for high-performance separations. This progressive crosslinking approach where multiple crosslinking reaction occurs at different temperature ranges has great potentials for making ultra-selective membranes for H_2_ recovery and CO_2_ capture. This concept is expected to work on other commercially available polymers, such as polysulfone, polyimides, etc., which are currently under development in our lab.

## Methods

### Preparation of membranes

Dense membranes were prepared by solution casting of filtered equimolar PIM-BM and Tröger’s Base (TB) in chloroform on a clean glass substrate. After the solvent was slowly evaporated in 2 days, the dry free-standing membranes were obtained and exposed to methanol soaking overnight and further dried in a vacuum oven at 70 °C for 24 h. The thickness of the PIM-BM/TB membrane was about 50 μm (±10 μm).

### Thermal analyses

Thermal analyses of PIM-BM/TB fresh and thermally treated membranes were performed in a TGA to study the thermal degradation under nitrogen atmosphere. Polymer membranes were dynamically heated from room temperature to 100 °C at 5 °C/min and held for 30 min then to 800 °C at 5 °C/min under nitrogen atmosphere. TG-MS were performed in TGA Q50 V20.10 Build 36 under 200 ppm O_2_ balanced with nitrogen.

### Thermal crosslinking treatments

The membranes of PIM-BM/TB were crosslinked using a CenturionNeytechQex vacuum furnace under 200 ppm O_2_ balanced with nitrogen. The vacuum furnace was swept for 60 min, then the temperature was raised between 80 and 300 °C at a rate of 3 °C/min and held for a period of 5–20 h. After the thermal crosslinking treatment process, the membranes were cooled at a rate of 3 °C/min in the furnace to room temperature for further studies. The membrane was labeled as “XPIM-BM/TB-temperature (h)”, for example, XPIM-BM/TB-80 °C-20 h.

### Characterization

XRD was used to study the change of *d*-spacing. The results were recorded on a Bruker AXS GADDS apparatus using Cu radiation with a wavelength of 1.54 Å (voltage: 40 kV, current: 30 mA). *d*-Spacing was computed following Bragg’s law (*d* = λ/2 sin θ). An XPS was utilized to monitor the chemical changes of PIM-BM/TB-fresh and thermally crosslinked PIM-BM/TB membranes. They were recorded on HSi spectrometer (Thermo Fisher ESCALAB 250 xi., England) using a monochromatic Al Kɑ X-ray source (1486.6 eV photons) at a constant dwell time of 100 ms and a pass energy of 40 eV under full vacuum. The anode voltage and anode current were 15 kV and 10 mA, respectively. All core-level spectra were obtained at a photoelectron take-off angle of 90° with respect to the sample. To compensate for surface charging effects, all binding energies (BE’s) were referenced to the C1s hydrocarbon peak at 284.8 eV. Surface elemental stoichiometries were determined from the peak area ratios and were accurate to within ±5%.

SEM analysis of membranes was performed using a Hitachi S5500 microscope. The polymer films were fractured and coated with a thin layer of gold. Determination of polymer molecular weights were accomplished using gel permeation chromatography (GPC, Shimadzu LC-20A) with Ultrastyragel columns and tetrahydrofuran (THF) as the eluent flowing at a rate of 1 mL/min. The FTIR measurements were performed using an attenuated total reflection mode (FTIR-ATR), with a Perkin-Elmer Spectrum 2000 FTIR spectrometer. Each sample was scanned 32 times. Wide-angle X-ray scattering was performed with a DX-2700 machine operated at 30 mA and 40 kV using Cu Kα radiation with a step of 0.03 per second. Tensile tests of polymer films were carried out at Instron-1211 (Instron Co., USA) mechanical testing instrument at a crosshead speed of 1 mm/min. Polymer films were cut into thin slices with an effective length of 10 cm and a width of 1 cm, with the accurate value determined from high-resolution photos and calibrations from known length. The average value of Young’s modulus was derived from the initial slope. The tensile strength at break and elongation at break were also measured. The positron annihilation experiments were conducted by using a fast-fast coincidence PALS. A ^22^Na source was used as positron source. The activity of the ^22^Na source is about 10 mCi. Kapton film is used to encapsulate dry ^22^Na source. The membranes were cut into 1 cm × 1 cm slice, the thickness of test slice was around 1.5 mm. Two slices of the same sample sandwiched a 20 μCi positron source (^22^Na), which was sealed with two thin Kapton membranes of 7 μm. The positron lifetime spectrum of single-crystal Ni was used as a reference in order to subtract the source components of positron annihilation in Kapton membranes and ^22^Na. The positron lifetime (τ) is obtained by the time difference between the emission of the birth γ ray (1.28 MeV) and the annihilation photon (0.511 MeV).

### Gas permeation

Pure gas permeation tests were carried out at a temperature of 35 °C with feed pressures up to 500 psi using a constant-volume variable-pressure apparatus. The mixed gas permeation properties were measured in the same membrane cell using the same constant-volume variable-pressure apparatus. The membrane was exposed to certified gas mixtures of CO_2_/CH_4_ (50/50 vol%) with feed pressures up to 500 psi at 35 °C. The gas compositions were analyzed by a gas chromatograph (GC-7820A, Agilent).

### Molecular simulation

The molecular dynamics (MD) simulation was constructed by the Forcite module in Materials Studio software package (Accelrys Inc., CA, USA). In one cubic simulation box, four polymer chains (2 PIM-BM-70% polymer chains and 2 TB polymer chains) with 10 repeating units were constructed. The initial density is 0.5 g/cm^3^ and the target density is 1.177 g/cm^3^ before crosslinking. The force field was PCFF. The Berendsen algorithm with a decay constant of 0.1 ps was used to control the temperature and pressure of each box. The specific procedures before crosslinking are as follows: (1) energy minimization; (2) 50 ps NVT-MD simulation at 600 K; (3) 100 ps NPT-MD simulation at 600 K at 1 GPa; (4) 100 ps NPT-MD simulation at 298.15 K at 1 GPa; (5) 100 ps NPT-MD simulation at 298.15 K at 0.1 MPa; and (6) 50 ps NVT-MD at 298.15 K. The Ewald summation method was used to calculate the non-bonded interactions with an accuracy of 0.001 kcal/mol.

The final equilibrium structure was used to crosslink Br with N or C atoms, and the cubic simulation box with crosslinked polymers was used as the initial structure for molecular dynamics simulation. The specific procedures after crosslinking are as follows: (1) 100 ps NPT-MD simulation at 298.15 K at 2 GPa; (2) 100 ps NPT-MD simulation at 298.15 K at 0.1 MPa; and (3) 50 ps NVT-MD simulation at 298.15 K. The force field and other parameters are the same as the ones used before crosslinking.

## Supplementary information

Supplementary information.

## Data Availability

The data that support the findings of this study are available from the corresponding author upon reasonable request.
