# Supplementary material for: Ultra-selective molecular-sieving gas separation membranes enabled by multi-covalent-crosslinking of microporous polymer blends
Source: Nat Commun. 2021 Oct 22;12:6140. doi: 10.1038/s41467-021-26379-5 (PMC8536662; doi:10.1038/s41467-021-26379-5)
Supplement: Supplementary file 1 — Supplementary information. [file 41467_2021_26379_MOESM1_ESM.pdf]

Supplementary Information

Ultra-selective molecular-sieving gas separation membranes enabled by multi-covalent-crosslinking of microporous polymer blends

Xiuling Chen[1,2#], Yanfang Fan[3#], Lei Wu[1], Linzhou Zhang,[3] Dong Guan,[3] Canghai Ma[4*], Nanwen Li[1*]

[1] State Key Laboratory of Coal Conversion, Institute of Coal Chemistry, Chinese Academy of Sciences, Taiyuan 030001

[2] Hubei Key Laboratory of Radiation Chemistry and Functional Materials, Hubei University of Science and Technology, Xianning 437100, China

[3] State Key Laboratory of Heavy Oil Processing, College of Chemical Engineering and Environment, China University of Petroleum-Beijing, Beijing, 102249, China

[4] State Key Laboratory of Fine Chemicals, Research and Development Center of Membrane Science and Technology, School of Chemical Engineering, Dalian University of Technology, Dalian, Liaoning, 116024, China

[#] X. Chen, and Y. Fan are equally contributed to this work.

cma@dlut.edu.cn (C.Ma); *linanwen@sxicc.ac.cn*

1. Supplemental experimental procedures

1.1 Synthesis of PIM-BM-x and TB

Synthesis of polymer PIM-M. The PIM-M was synthesized according to the literature reported.[1] The flask was swept with argon for 30 min to remove the dissolved moisture and air. Equal molar ratio of 4,4'-dimethyl-3,3,3',3'-tetramethyl-2,2',3,3'-tetrahydro-1,1'-spirobi[indene]-5,5',6,6'-tetraol (TTSBI-Me, 10 mmol) and 2,3,5,6-tetrafluoroterephalonitrile (TFTPN, 10 mmol) with a stoichiometric amount of anhydrous K_2CO_3 (2.5 times compared to monomers) were dissolved in anhydrous NMP and toluene (volume ratio of 3:1) and the mixture was quickly heated to 155 °C for 4 h. After cooling to the room temperature, the solids were filtrated and washed with methanol and water. The yellow solid was washed in a 0.1wt.% HCl aqueous solution to remove the catalyst. The product was then filtered, washed with deionized water, and washed again with methanol for removing the residual HCl. The collected yellow polymer was dried under vacuum at 80 °C for 24 h prior to use (Supplementary Fig. 1).

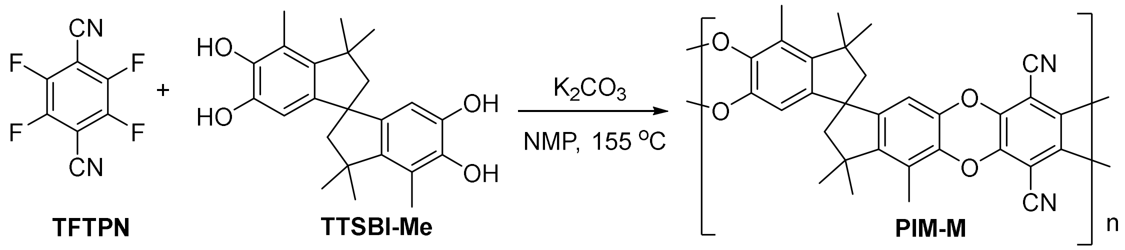

Supplementary Fig. 1 Reaction scheme for PIM-M synthesis

Synthesis of polymer PIM-BM-70%[1]**.** The PIM-BM-70% was synthesized through free-radical bromination of PIM-M using different ratios of *N*-bromosuccinimide (NBS) and azodiisobutyronitrile (AIBN). PIM-M (4.88 g, 10 mmol) was dissolved in chlorobenzene (100 mL) under an inert atmosphere, then NBS (7 mmol) and AIBN (0.7 mmol $n_{NBS} : n_{AIBN} = 10 : 1$) were added to the reaction solution and refluxed at 135 °C for 3-4 h. After cooling, the mixture was poured into methanol (500 mL) to obtain a yellowish powder. The polymer product was washed for 4-5 times using methanol and dried at 70 °C for 24 h (Supplementary Fig. 2). The results of ^{1}H NMR were shown in Supplementary Fig. 3. The molecular weight was estimated with the results of Mw=56721.

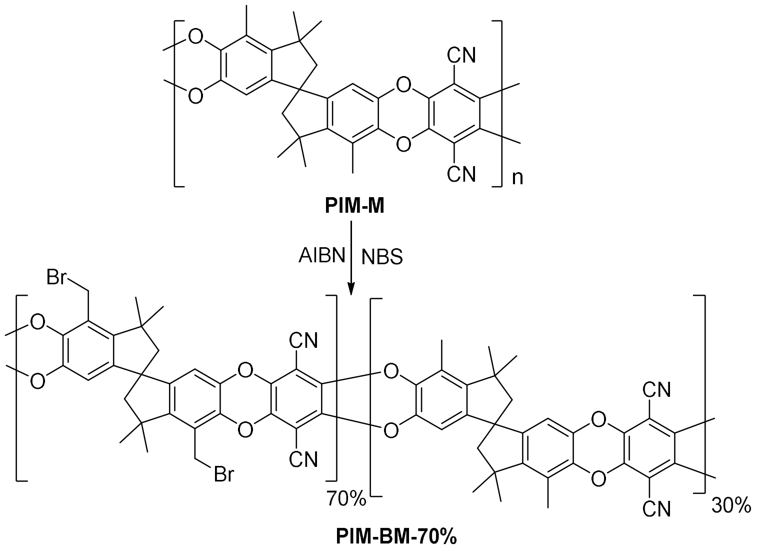

Supplementary Fig. 2 Reaction scheme for PIM-BM-70% synthesis

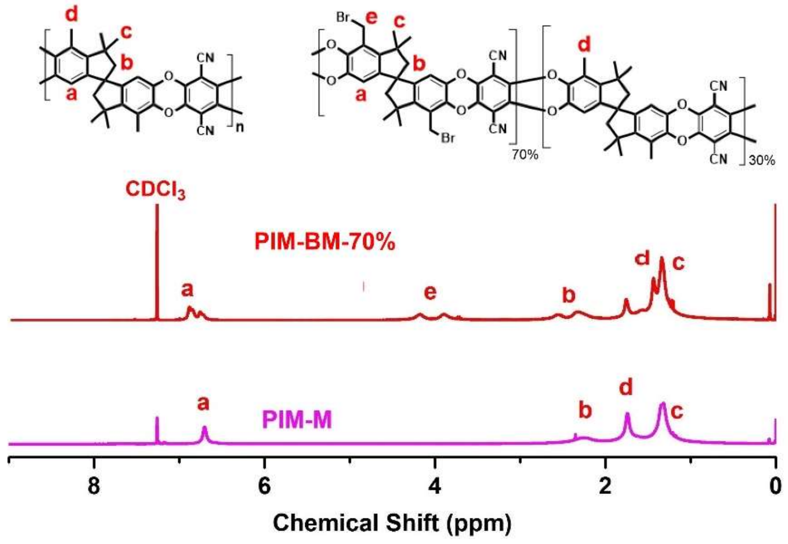

Supplementary Fig. 3 ^{1}H NMR of PIM-M and PIM-BM-70%

Synthesis of Tröger's Base. Tröger's Base polymer was prepared according to the previous report.[2] 4,4'-diamino-3,3'-dimethylbiphenyl (10g, 47.169 mmol) and diethoxymethane (21 mL, 235.8 mmol) were added into the three round bottom flask at 0 ºC. After being fully dissolved, trifluoroacetic acid (80 mL) was added dropwisely into the reaction mixture at 0 ºC. After the reaction the mixture was further stirred at a room temperature for 4 days. The mixture was poured into ammonia aqueous solution. The solid product was filtered, and purified by redissolving and precipitating three times in chloroform and methanol (Supplementary Fig. 4). The result of ^{1}H NMR was shown in Supplementary Fig. 5. The molecular weight was estimated with the results of Mw=107900.

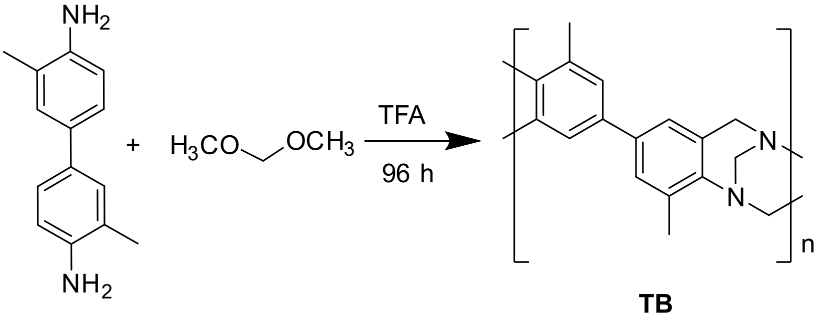

Supplementary Fig. 4 Reaction scheme for the synthesis of TB

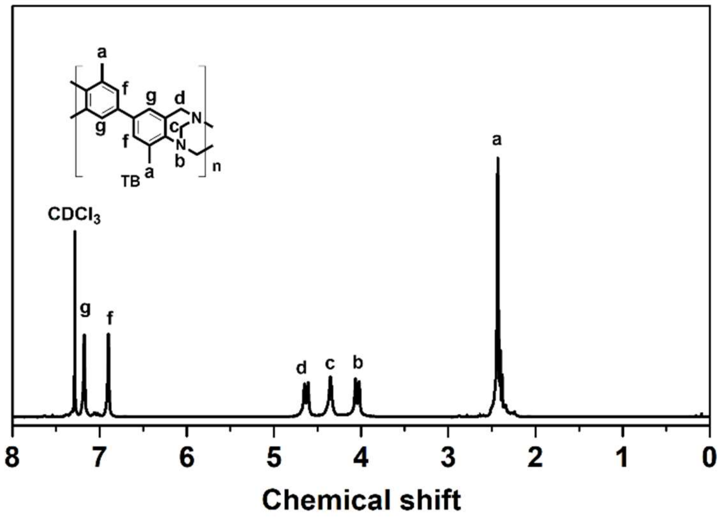

Supplementary Fig. 5 ^{1}H NMR of TB

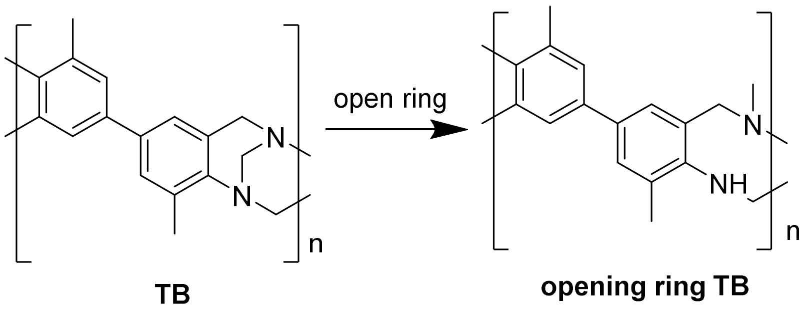

Supplementary Fig. 6 Reaction scheme for opening ring TB

1.2 Possible mechanism as shown in Supplementary Fig. 7-Supplementary Fig. 9

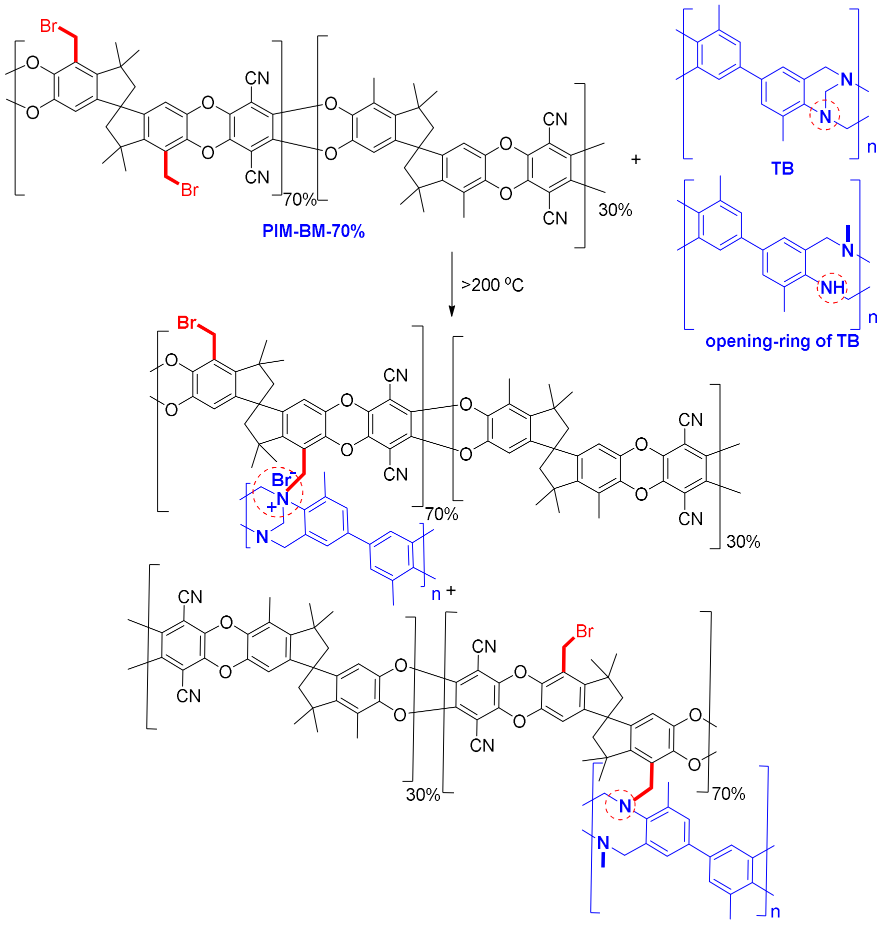

Supplementary Fig. 7 Reaction scheme of tertiary amine with bromomethyl groups and ring opening reaction of TB

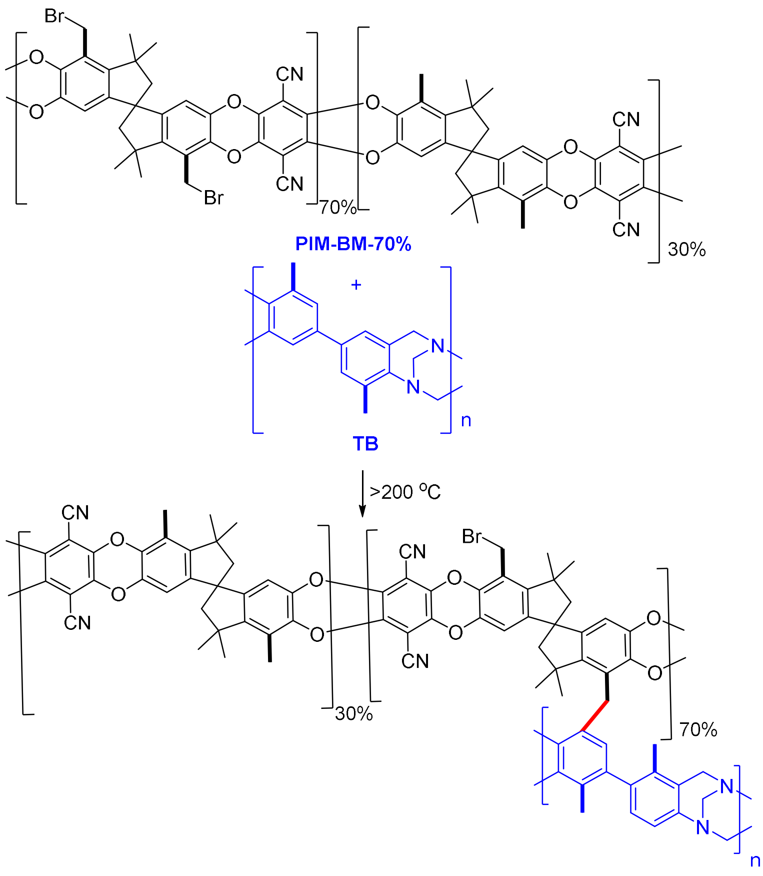

Supplementary Fig. 8 Reaction scheme of nucleophilic reactions between C-Br bonds and phenyl rings in TB

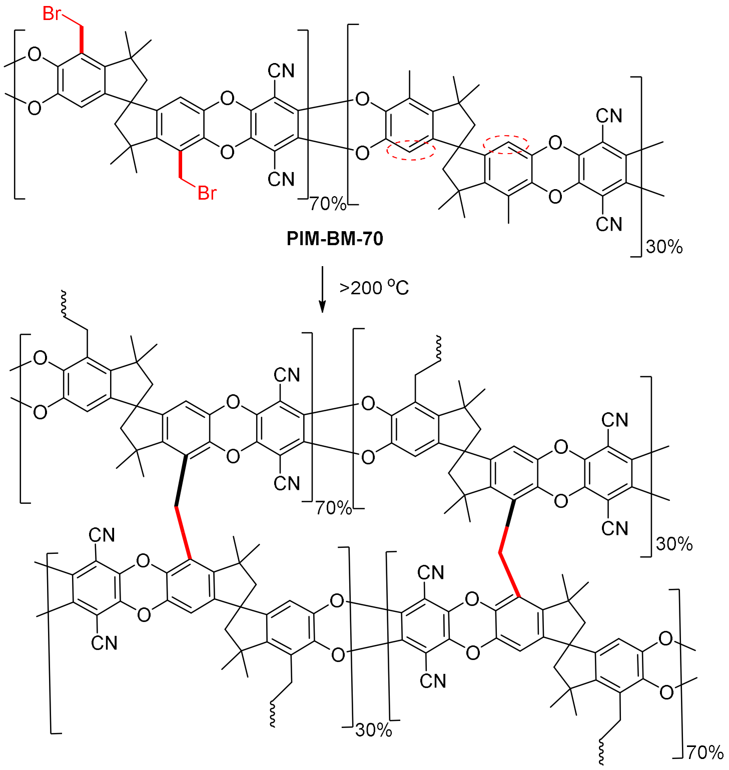

Supplementary Fig. 9 The reactions between C-Br bonds and phenyl rings in PIM-BM-70%

Supplementary Table 1 Simulation results for crosslinked membranes

Sample	Simulated density (g/cm^3)	Experimental density (g/cm^3)	FFV	
			FFV (H_2)	FFV (CO_2)
PIM-BM/TB	1.141	1.177	0.228	0.194
PIM-BM	1.129	1.090	0.341	0.313
120-°C-20 h	1.145	1.107	0.225	0.193
200-°C-20 h	1.159	1.136	0.214	0.189
250-°C-10 h	1.169	1.190	0.201	0.178
300-°C-5 h	1.214	1.223	0.187	0.163

2. Characterization of polymer membranes

2.1 Photo of XPIM-BM/TB polymer

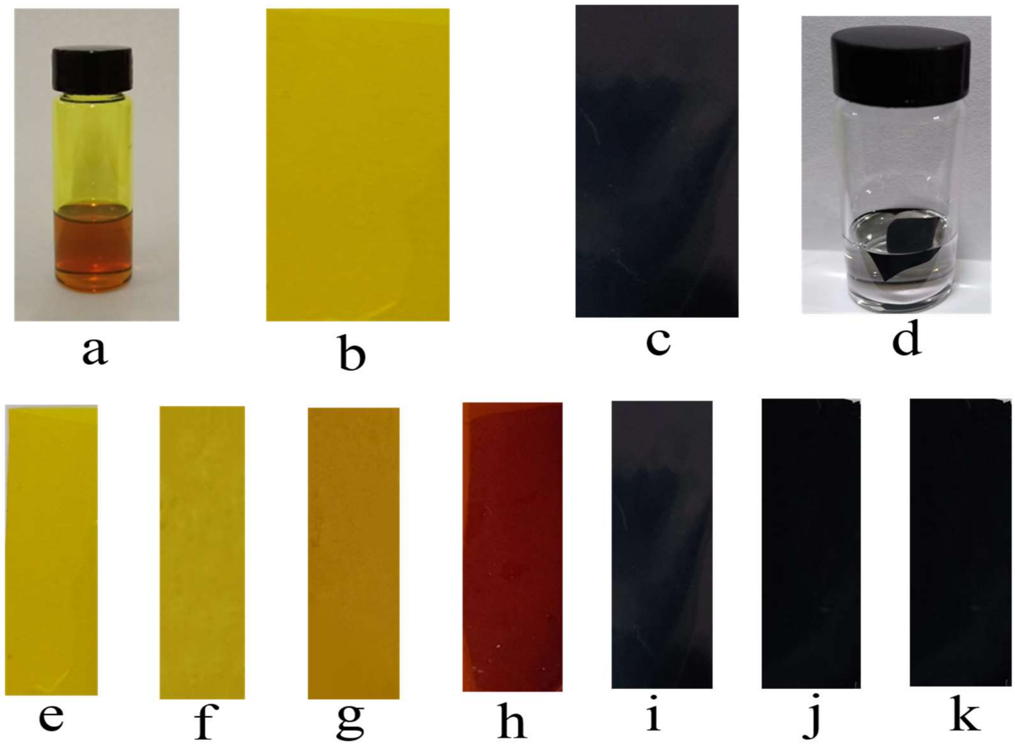

Supplementary Fig. 10 Characterization of polymer membranes. (a) Photo of XPIM-BM/TB polymer solution in chloroform. (b) Photo of as-prepared XPIM-BM/TB membrane. (c) Photo of thermal-crosslinked (300 °C-5 h) XPIM-BM/TB membrane. (d) Photo of thermal-crosslinked (300 °C-5 h) XPIM-BM/TB membrane solubility test in chloroform. (e) Photo of as-prepared XPIM-BM/TB membrane without thermal management. (f) Photo of XPIM-BM/TB membrane at 80 °C for 20 h. (g) Photo of XPIM-BM/TB membrane at 120 °C for 20 h. (h) Photo of XPIM-BM/TB membrane at 200 °C for 20 h. (i) Photo of XPIM-BM/TB membrane at 250 °C for 20 h. (j) Photo of XPIM-BM/TB membrane at 300 °C for 2 h. (k) Photo of XPIM-BM/TB membrane at 300 °C for 5 h.

2.2 XPS of fresh and the thermally cross-linked XPIM-BM/TB membranes

An X-ray photoelectron spectra of membranes. Supplementary Fig 6 shows Br_{3d} XPS spectrum of the fresh PIM-BM/TB and thermally cross-linked XPIM-BM/TB membranes. The fresh PIM-BM/TB membranes reveals a symmetrical Br_{3d} core signal with the maximum peak at 70.4 eV which is ascribed to the C-Br covalent bonded.[5-9]

The crosslinking degree is calculated as follows (Supplementary Table 2): 120 °C-20 h: degree of alkylation reaction is not detected, degree of quaternary is 12% (area of the bromide ion / area of the original film). 200 °C-20 h: degree of alkylation reaction is not detected, degree of quaternary is 18% (area of the bromide ion / area of the original film). 250 °C-10 h: degree of alkylation reaction is 22% (area of the bromine lost / area of the original film), degree of quaternary is 25% (area of the bromide ion / area of the original film). 300 °C-5 h: degree of alkylation reaction is 40% (area of the bromine lost / area of the original film), degree of quaternary is 45% (area of the bromide ion / area of the original film). 300 °C-10 h: degree of alkylation reaction is 41% (area of the bromine lost / area of the original film), degree of quaternary is 57% (area of the bromide ion / area of the original film).

Supplementary Table 2 The calculated degree of crosslinking XPIM-BM/TB[a]

Membranes	Degree of quaternary	Degree of alkylation reaction	Total degree
RT	0	0	0
120-°C-20 h	12%	0	12%
200-°C-20 h	18%	0	18%
250-°C-10 h	25%	22%	47%
300-°C-5 h	40%	45%	85%
300-°C-10 h	57%	41%	98%

[a] the calculated based on XPS survey spectrum of Br

2.3 TGA and TG-MS of membranes

TG-MS and TGA results are shown in Supplementary Fig. 11- Supplementary Fig. 12. The results revealed that pristine PIM-BM-70% and TB polymer blends shows an initial mass loss at 226 °C, indicating the occurrence of thermal cross-linking of C-Br bonds and phenyl rings through alkylation reaction with the loss of HBr. The membranes treated at temperatures of above 250 °C are thermally stable with initial mass loss at temperature above 320 °C in N_2 atmosphere. This result reflects the majority of C-Br bonds have been consumed through alkylation reaction upon thermally treated at 250 °C and 300 °C.

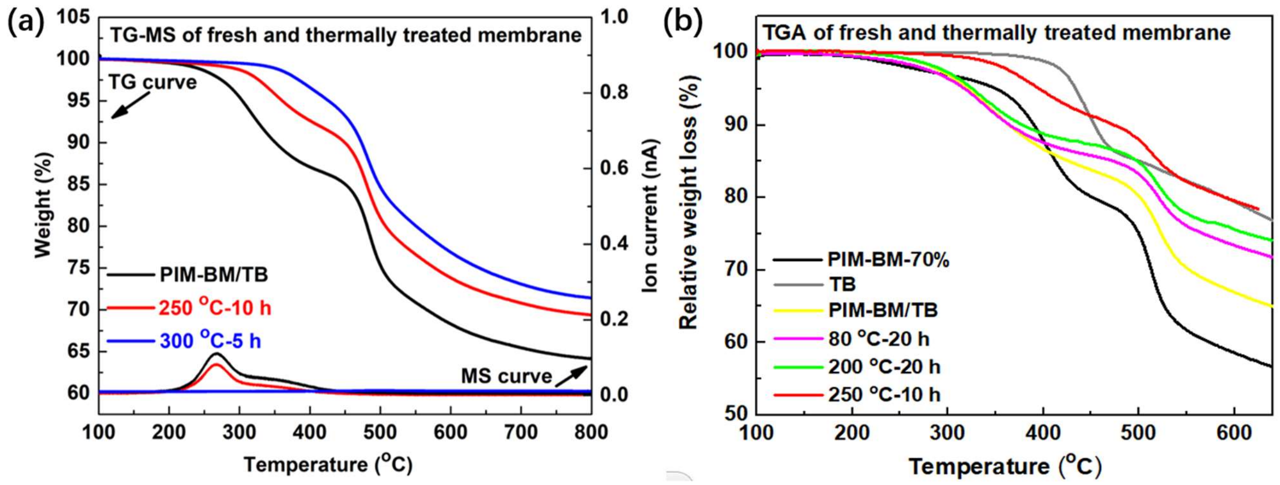

Supplementary Fig. 11 (a) TG-MS of PIM-BM/TB and the thermally cross-linked XPIM-BM/TB, **(b)**TGA curves of PIM-BM-70%, TB and PIM-BM/TB and the thermally cross-linked XPIM-BM/TB

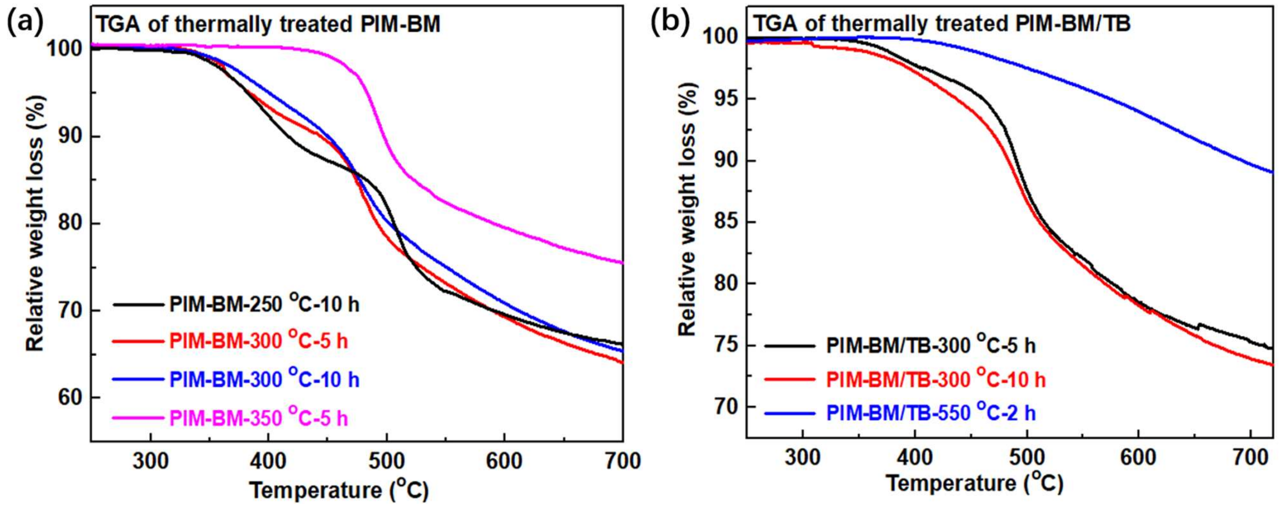

Supplementary Fig. 12 (a) TGA curve of thermally treated PIM-BM, **(b)** TGA curve of thermally treated PIM-BM/TB

2.4 IR of membranes

Supplementary Fig. 13-Supplementary Fig. 14 depict the ATR-IR spectra for the original PIM-BM-70%, TB, PIM-BM/TB, and thermally treated XPIM-BM/TB. The ATR-IR spectrum of the crosslinked and uncrosslinked PIM-BM-Br-70 have been investigated, As shown in Supplementary Fig. 13a, the peak around 2247 cm^{-1} which is attributed to nitrile stretching (C≡N) kept intact after thermal crosslinking, indicating the non-reaction between the CN groups.[9] However, the characteristic peak of C-Br stretching vibration at 660 cm^{-1} has been weakened after crosslinking.[10] This result indicates that the thermal crosslinking of PIM-BM-70% has occurred by the loss of HBr to form the methylene groups. Moreover, the crosslinking mechanism has been further confirmed by TGA-MS, in which the intensities of atomic mass, m/z is determined to be 81 that could be attributed to the HBr emission (Figure 11a). Supplementary Fig. 13b shows TB-fresh and thermally treated TB, the results show that TB membrane gradually open ring with the temperature increasing. Peak at 1668 cm^{-1} is attributed to –C-N of tertiary amine, peak at 1608 cm^{-1} is attributed to –C-N of secondary amine[11-12], 3360 cm^{-1} is attributed to –N-H. Supplementary Fig. 14 shows

PIM-BM/TB-fresh and thermally treated XPIM-BM/TB, the results shows that nitrile stretching (C≡N) also remains intact after thermal crosslinking, C-Br stretching vibration at 860 cm^{-1} has been weakened after crosslinking and C-Br stretching vibration at 660 cm^{-1} disappears when the membrane is treated at 300 ^{o}C for 5 h, ascribed to alkylation reaction and quaternary amination reactions (reactions of tertiary amine with bromomethyl groups) between PIM-BM-70% and TB.

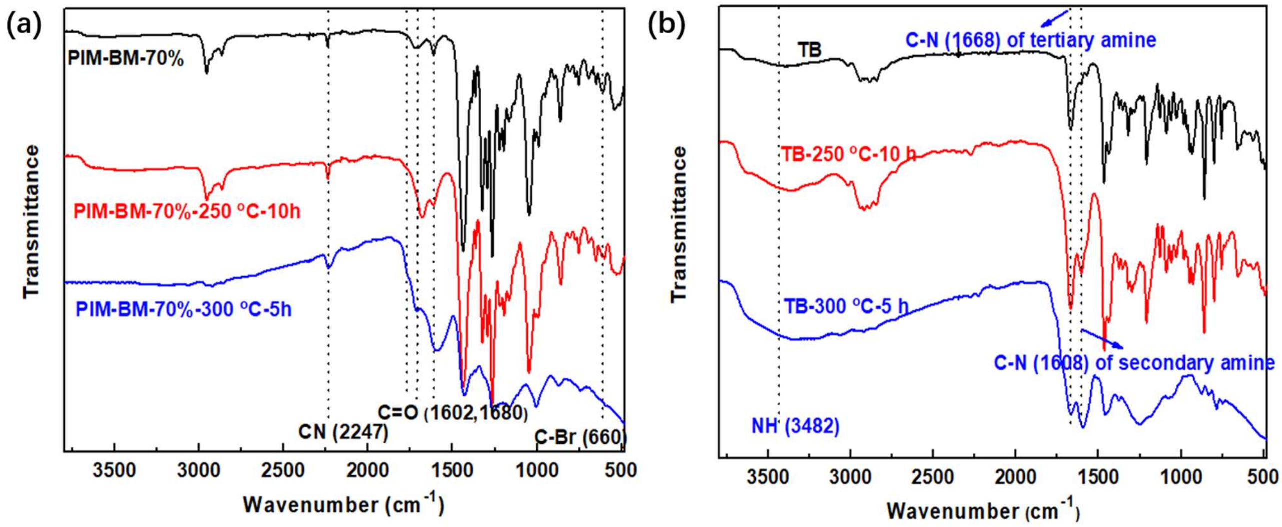

Supplementary Fig. 13 ATR-IR of fresh and thermal treated **(a)** PIM-BM-70%, **(b)** TB

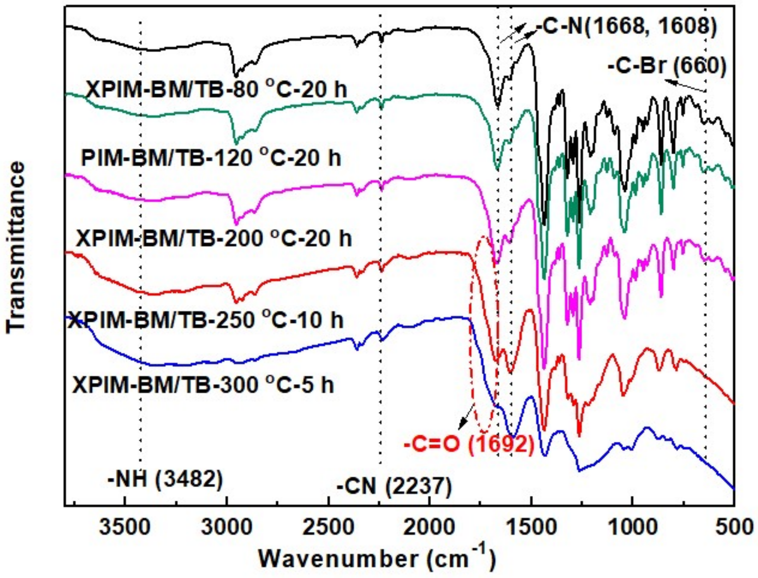

Supplementary Fig. 14 ATR-IR of PIM-BM/TB and thermal treated XPIM-BM/TB

2.5 SEM of membranes

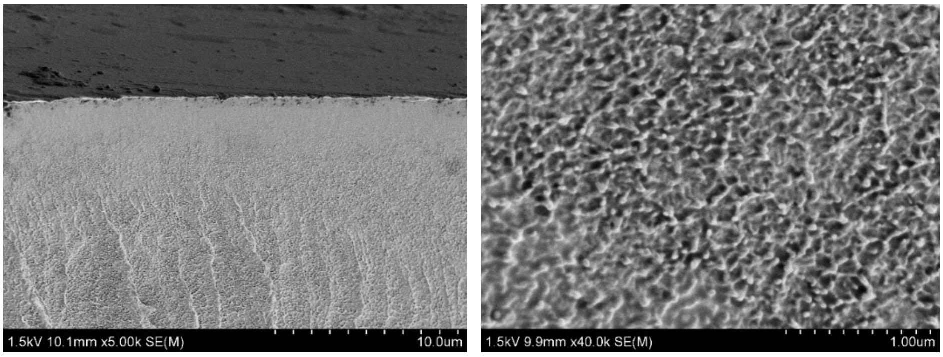

Cross-sectional SEM images of PIM-BM/TB fresh polymer films

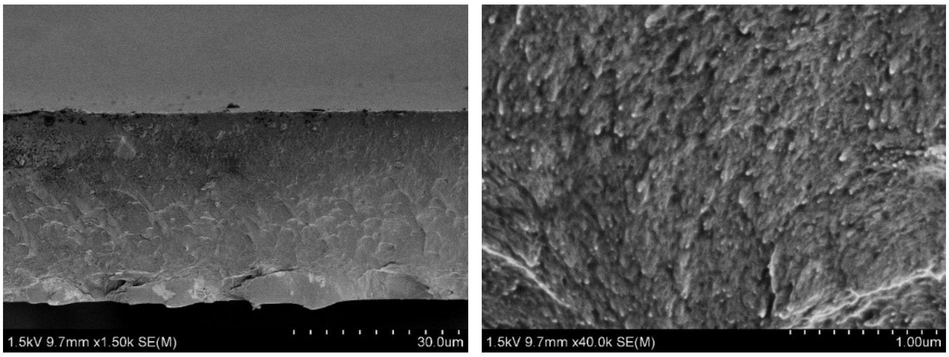

Cross-sectional SEM images of XPIM-BM/TB-200 ^{o}C-20 h polymer films

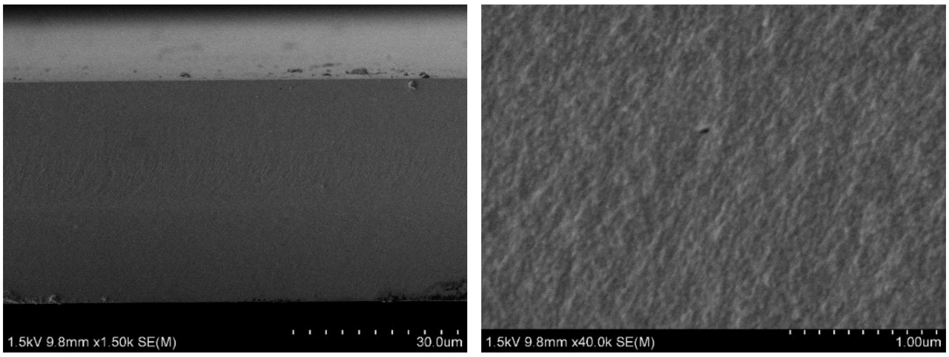

Cross-sectional SEM images of XPIM-BM /TB-250 ^{o}C-10 h polymer films

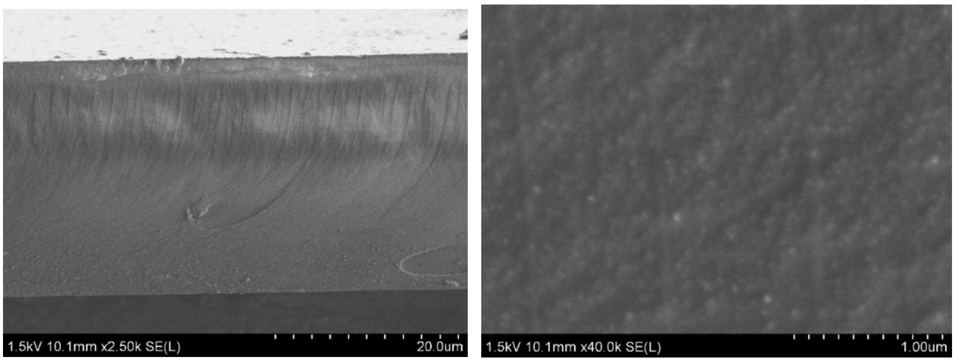

Cross-sectional SEM images of XPIM-BM/TB-300 ^{o}C-5 h polymer film

Supplementary Fig. 15 Cross-sectional SEM images of PIM-BM/TB fresh and XPIM-BM/TB

2.6 Raman spectra of membranes

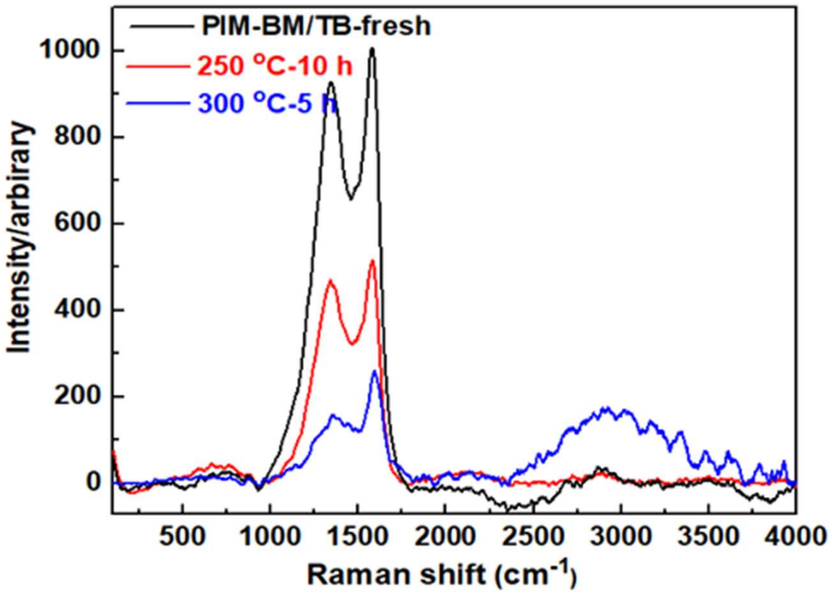

Supplementary Fig. 16 Raman spectra of PIM-BM/TB treated under varied conditions

2.7 Upper bounds of membranes

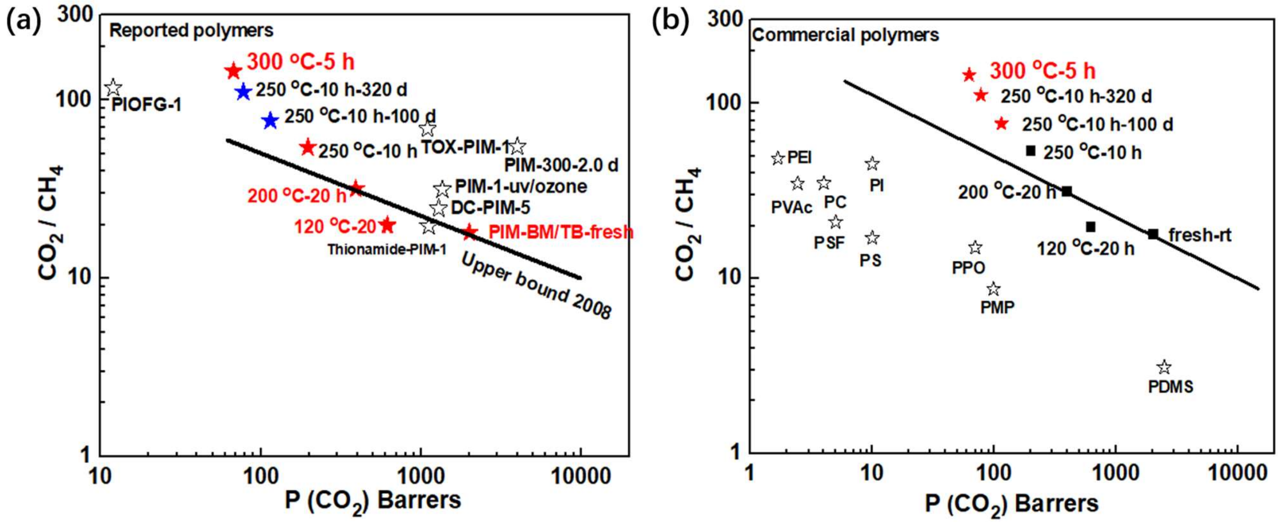

Supplementary Fig. 17 Robeson plot of CO_2/CH_4 selectivity versus CO_2 permeability **(a)** reported polymers, **(b)** commercial polymers

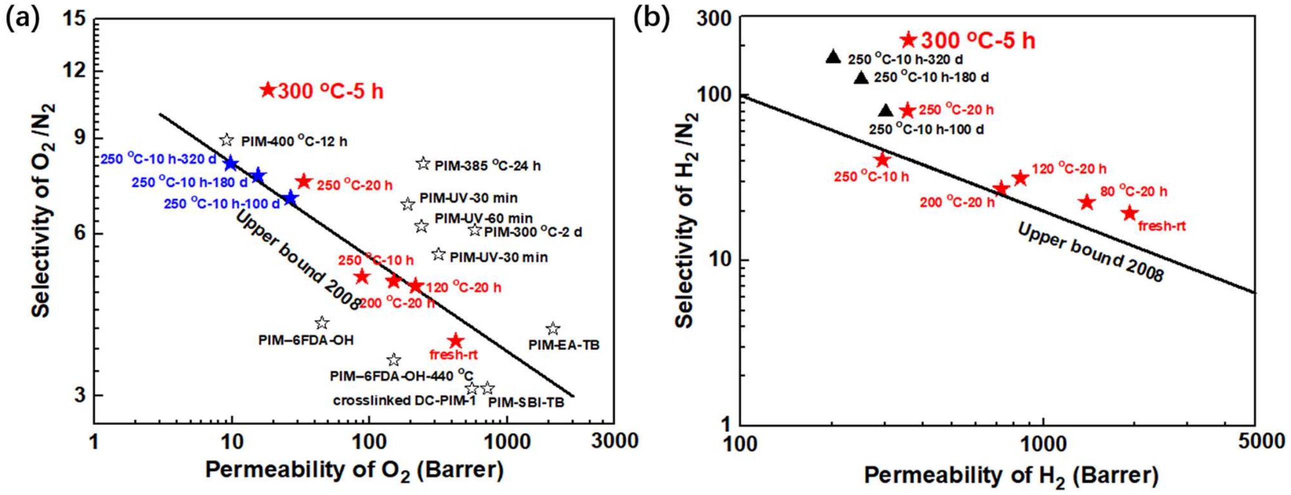

Supplementary Fig. 18 Robeson plot of **(a)** H_2/N_2 selectivity versus H_2 permeability, **(b)** O_2/N_2 selectivity versus O_2 permeability

2.8 Mechanical properties and XPS of membranes

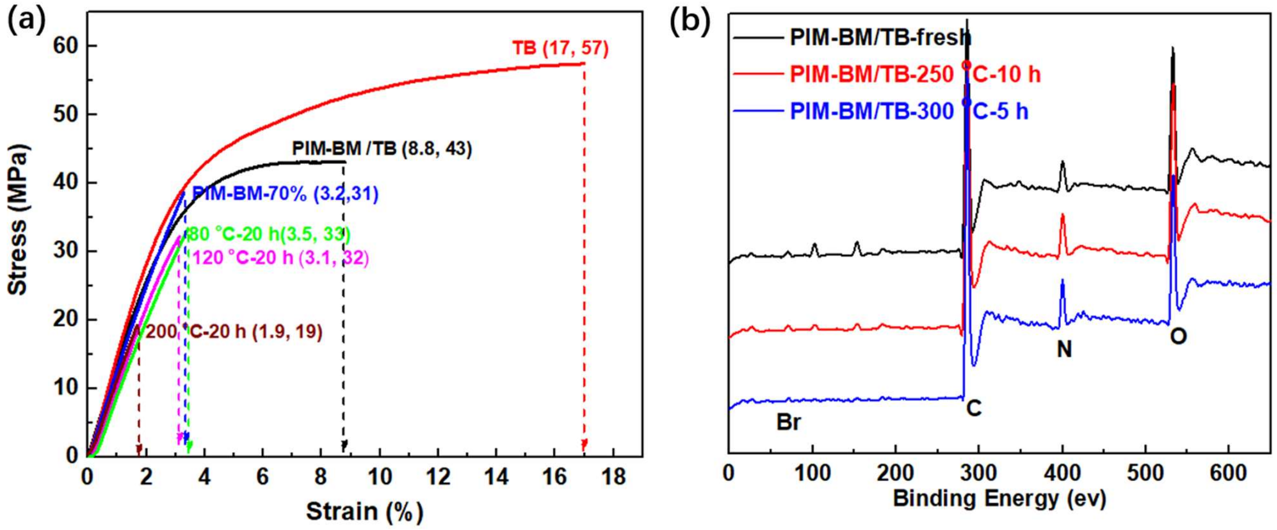

Supplementary Fig. 19 (a) Stress-strain curve of PIM-BM-70%, TB and XPIM-BM /TB, **(b)** XPS of PIM-BM/TB and crosslinked PIM-BM/TB

2.9 Relative changes in permeability and selectivity for various gases

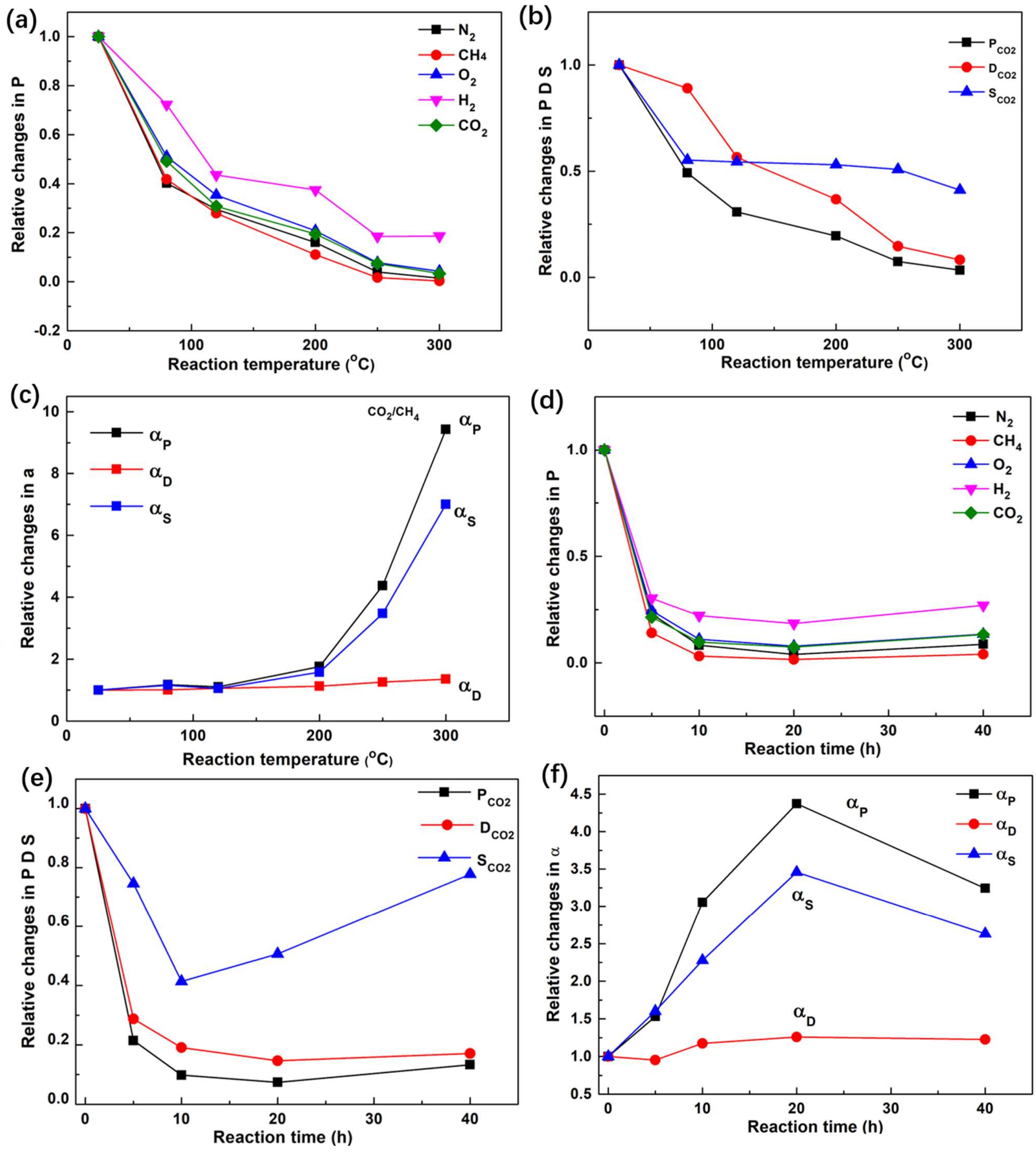

Supplementary Fig. 20 Relative changes in P for various gases and selectivities of gas pairs. **(a)** All gas relative changes in permeability at 80-300 °C, **(b)** CO_2 relative changes in P D S at 80-300 °C, **(c)** CO_2/CH_4 relative changes in a at 80-300 °C, **(d)** All gas relative changes in permeability of 250 °C at different time, **(e)** CO_2 relative changes in P D S of 250 °C at different time, **(f)** relative changes of α_P α_D α_S of 250 °C at different time.

2.10 Comparison of PIM-BM/TB gas permeability

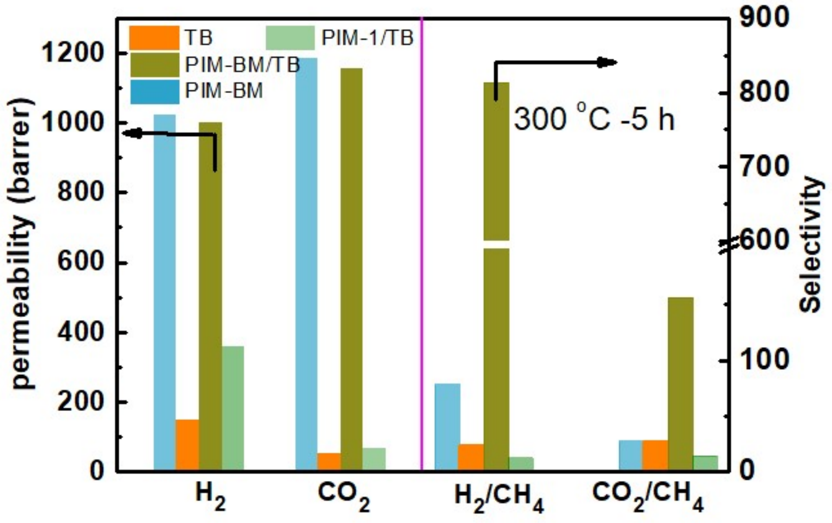

Supplementary Fig. 21 Comparison of PIM-BM/TB gas permeability and H_2/CH_4 and CO_2/CH_4 selectivities with TB, PIM-BM (70%), PIM-1 (treated at 300 °C 5 h, 200 ppm O_2)

Supplementary Table 3 Mechanical properties of membranes tested at room temperature

Membrane	Tensile strength [MPa]	Elongation at break [%]	Young's modulus [GPa]	
TB	55.5±3.6	17.1±0.3	1.65±0.2	This work
PIM-BM-70%	31.5±1.9	3.2±0.1	1.66±0.1	This work
PIM-BM/TB	42.5±3.3	8.8±0.1	1.24±0.1	This work
XPIM-BM/TB-80 °C-20 h	33.4±1.9	3.5±0.2	1.20±0.2	This work
XPIM-BM/TB-120 °C-20 h	32.4±2.6	3.1±0.2	1.32±0.1	This work
XPIM-BM/TB-200 °C-20 h	19.4±0.9	1.9±0.1	1.05±0.1	This work
XPIM-BM/TB-300 °C-5 h	22.0±1.1	0.97±0.1	1.01±0.1	This work
PIM-1	47.5±2.3	14.3	1.43±0.15	13
TOX-PIM-1 385°C 1 mbar 8 h	56.5±2.8	7.1	1.28±0.37	13
TOX-PIM-1 385°C 1 mbar 12 h	60.0±3.0	5.8	1.45±0.05	13
spiroTR-PBO-6F	82.3±1.3	20.0±4.0	n.a.	14
PIOFG-1	83	3.1	n.a.	15
TR-1-350	87	3.8	n.a.	15
TR-1-400	95	3.5	n.a.	15
TR-1-450	98	3.9	n.a.	15

3. Gas transport properties of XPIM-BM-TB

3.1 Gas transport properties of PIM-BM/TB blending membranes

Supplementary Table 4 Gas permeabilities and ideal selectivities of PIM-BM with a bromomethylation degree of 70%, TB and PIM-BM/TB blending membranes

Polymer	P (Barrer) [a]					Selectivity [b]				
	N_2	CH_4	O_2	H_2	CO_2	CO_2/N_2	CO_2/CH_4	O_2/N_2	H_2/N_2	H_2/CH_4
PIM-BM-70%	407	575	1048	3531	6047	14.8	10.6	2.6	8.7	6.2
TB	6.3	7	34	280	134	21.2	19.1	5.4	44.4	40.0
PIM-BM/TB =10:1	226	307	790	2850	4223	18.7	13.8	3.5	12.6	9.2
PIM-BM/TB =10:3	209	252	695	2478	3644	17.4	14.5	3.3	11.8	9.8
PIM-BM/TB =10:5	142	189	501	1904	2532	17.8	13.4	3.5.	13.4	10.1
PIM-BM/TB =10:7	131	164	471	1886	2327	17.8	14.2	3.6	14.4	11.5
PIM-BM/TB =1:1	110	112	423	1925	2007	18.2	17.9	3.8	17.5	17.2

[a] Permeability coefficients measured at 35 °C and 50 psi feed pressure. 1 barrer = 10^{-10} [cm^3 (STP) cm]/(cm^2 s cmHg)

[b] Ideal selectivity $\alpha = (P_a)/(P_b)$

Supplementary Table 5 Gas permeabilities and ideal selectivities of PIM-BM-70% and TB at different temperature under the nitrogen flow with 200 ppm O_2

Polymer	P (Barrer) [a]					Selectivity [b]				
	N_2	CH_4	O_2	H_2	CO_2	CO_2/N_2	CO_2/CH_4	O_2/N_2	H_2/N_2	H_2/CH_4
PIM-BM-70%	407	575	1048	3531	6047	14.8	10.5	2.6	8.7	6.2
PIM-BM-70%-250 °C-10 h	51	70	183	748	1229	24.1	17.6	3.6	14.6	10.7
PIM-BM-70%-300 °C-5 h	53	42	212	1003	1158	21.8	27.6	4.0	18.9	23.9
Troger's base	6.3	7.0	34	280	134	21.3	19.1	5.4	44.4	40.0
Troger's base-250 °C-10 h	2.0	1.25	11	114	37	18.1	29.2	5.5	56.3	91.0
Troger's base-300 °C-5 h	3.5	1.9	16	149	52	14.9	27.4	4.6	42.6	78.4
PIM-BM/TB = 1:1-rt	110	112	423	1925	2007	18.2	17.9	3.8	17.9	17.2
PIM-1-400 °C-12 h[13]	1.0	0.32	9.1	180.5	35.6	34.9	110	8.95	177	557
PIM-1/TB	180	219	624	2043	2634	14.6	12.0	3.5	11.4	9.3
PIM-1/TB-250 °C-10 h	91	107	338	1193	1412	15.5	13.1	3.7	13.1	11.7
PIM-1/TB-300 °C-5 h	71	86	281	1023	1186	16.7	13.8	3.9	14.4	11.9

[a] Permeability coefficients measured at 35 °C and 50 psi feed pressure. 1 barrer = 10^{-10} [cm^3 (STP) cm]/(cm^2 s cmHg)

[b] Ideal selectivity $\alpha = (P_a)/(P_b)$

Supplementary Table 6 Gas permeabilities and ideal selectivities of thermally treated PIM-BM/TB under the nitrogen flow with 200 ppm O_2

Polymer	P (Barrer) [a]					Selectivity [b]				
	N_2	CH_4	O_2	H_2	CO_2	CO_2/N_2	CO_2/CH_4	O_2/N_2	H_2/N_2	H_2/CH_4
80 °C-20 h	44	47	216	1392	987	22.4	21.0	4.9	31.6	29.6
120 °C-20 h	33	31	150	839	618	18.7	19.9	4.5	25.4	27.1
200 °C-20 h	18	12	88	721	391	22.4	32.6	4.9	40.1	60.1
250 °C-5 h	25	16	104	582	431	17.2	26.9	4.2	23.3	36.4
250 °C-10 h	9	3.6	47	427	197	21.8	54.7	5.2	47.4	118.6
250 °C-20 h	4.4	1.8	33	356	149	33.9	82.7	7.5	80.9	197.7
250 °C-40 h	9.6	4.6	56.9	519	267	27.8	58.0	5.9	54.1	112.8
300 °C-2 h	9.4	4.0	58.8	455	218	23.2	54.5	6.3	48.4	113.8
300 °C-5 h	1.6	0.44	18	358	68	42.5	154.5	11.1	223.8	813.6
300 °C-10 h	15.7	5.9	97	739	366	23.3	62.0	6.1	47.1	125.3
550 °C-2 h[c]	9.1	3.2	29.1	351	170	18.7	54.8	3.2	38.6	119.1

[a] Permeability coefficients measured at 35 °C and 50 psi feed pressure. 1 barrer = 10^{-10} [cm^3 (STP) cm]/(cm^2 s cmHg).

[b] Ideal selectivity $\alpha = (P_a)/(P_b)$.

[c] N_2 atmosphere.

4. The plasticization resistance of the membranes

Supplementary Table 7 CO_2 plasticization resistance [a] and selectivity [b] of CO_2 / CH_4

	Total feed				Total feed		
Polymer	P (psi)	P(Barrer)	Selectivity	Polymer	P (psi)	P (Barrer)	Selectivity
	CO_2	CO_2	CO_2/CH_4		CO_2	CO_2	CO_2/CH_4
PIM-BM/TB	50	2007	18.9	80 °C-20 h	50	987.4	21.0
	100	1989	17.8		100	867	18.5
	150	1847	16.5		150	714	15.3
	250	1545	13.8		250	626.6	13.4
	350	1439	12.8		350	626.3	13.4
	450	1363	12.2		450	647.1	13.8
	500	1320	11.8		500	649.1	13.9
	Total feed				Total feed		
Polymer	P (psi)	P(Barrer)	Selectivity	Polymer	P (psi)	P (Barrer)	Selectivity
	CO_2	CO_2	CO_2/CH_4		CO_2	CO_2	CO_2/CH_4
200 °C-20 h	50	391.3	32.6	250 °C-10 h	50	197	54.7
	100	378.2	30.5		100	142.7	39.6
	150	364.8	29.4		150	128.4	35.7
	250	347.1	28.0		250	115.6	32.1
	350	330.1	26.6		350	105.3	29.3
	450	324.2	26.1		450	95.8	26.6
	500	293.2	23.6		500	89.9	25.0
	Total feed				Total feed		
Polymer	P (psi)	P (Barrer)	Selectivity	Polymer	P (psi)	P (Barrer)	Selectivity
	CO_2	CO_2	CO_2/CH_4		CO_2	CO_2	CO_2/CH_4
300 °C-2 h	50	218.4	54.5	300 °C-5 h	50	79	179.5
	100	205.1	51.3		100	67.6	154.5
	150	195.7	48.9		150	62	140.9
	250	162.8	40.7		250	60	136.4
	350	149.2	37.3		350	59.1	134.3
	450	138.6	34.7		450	58.3	132.5
	500	135.7	33.9		500	57.9	131.6

[a] Permeability coefficients measured at 35 °C and transform psi feed pressure. 1 barrer = 10^{-10} [cm^3 (STP) cm]/(cm^2 s cmHg)

[b] Ideal selectivity α = $(P_a)/(P_b)$

5. The aging of thermal cross-linking XPIM-BM/TB

Supplementary Table 8 The aging of thermal cross-linking XPIM-BM/TB (250 °C-10 h) at air for 0-320 day under 35 °C and 50 psi

	P (Barrer) [a]					Selectivity [b]				
Aged time	CH_4	N_2	O_2	CO_2	H_2	CO_2/CH_4	CO_2/N_2	O_2/N_2	H_2/CH_4	H_2/N_2
1 day	3.6	9.1	46.8	197	427.1	54.7	21.9	5.3	118.6	46.9
10 day	3.5	7.8	42.9	181.4	407.6	51.8	23.2	5.5	116.5	52.3
20 day	2.9	7.2	39.7	158.6	293.8	54.3	21.7	5.5	101.3	40.8
30 day	2.8	7.7	43.6	156.7	410.2	55.9	20.4	5.7	146.5	53.2
40 day	3.2	9.1	48.1	177.6	380.3	55.5	19.5	5.3	118.8	41.9
60 day	2.2	5.9	32.5	123.3	270.4	56.4	20.9	5.5	122.9	45.8
100 day	1.5	3.8	26.6	114.9	301.3	76.6	30.0	7.0	200.9	79.3
180 day	1.0	2.0	15.4	99.2	249.9	99.2	49.6	7.7	249.9	124.9
320 day	0.7	1.2	9.7	77.8	202.1	111.1	64.8	8.1	288.7	168.4

[a] Permeability coefficients measured at 35 °C and 50 psi feed pressure. 1 barrer = 10^{-10} [cm^3 (STP) cm]/(cm^2 s cmHg)

[b] Ideal selectivity $\alpha = (P_a)/(P_b)$

6. Diffusion coefficients and solubility coefficients

Supplementary Table 9 CO_2 and CH_4 diffusion coefficients and solubility coefficients for XPIM-BM-TB at different temperature

Polymer	diffusion coefficient (10^{-8} cm^2 s^{-1})			solubility coefficient (10^{-2} cm (STP) cm^{-3} cmHg)		
	CO_2	CH_4	$(\alpha)_D$	CO_2	CH_4	$(\alpha)_S$
PIM-BM-70%	10.7	2.2	4.9	753.6	348.6	2.2
PIM-BM-70%-250°C-10 h	5.4	0.98	5.5	394.6	159.2	2.5
PIM-BM-70% -300°C-5 h	2.1	0.36	5.8	551.4	116.7	4.7
TB-rt	21.3	4.3	5.0	6.3	1.6	3.9
TB-250 °C-10 h	11.9	2.7	4.4	5.0	0.78	6.4
TB-300 °C-5 h	7.1	1.1	6.5	7.3	1.7	4.3
nPIM-BM-70%:nTB=10:1	7.34	1.82	4	575.3	168.7	3.4
nPIM-BM-70%:nTB= 10:3	7.06	1.63	4.3	516.1	154.6	3.3
nPIM-BM-70%:nTB= 10:5	6.74	1.13	5.9	375.7	167.3	2.24
nPIM-BM-70%:nTB= 10:7	5.15	0.62	8.3	451.8	264.5	1.7
nPIM-BM-70%:nTB= 1:1	4.03	0.43	9.2	498.0	260.0	1.9
1:1-80 °C-20 h	3.59	0.38	9.4	275.0	123.2	2.3
1:1-120 °C-20 h	2.28	0.23	9.8	270.8	135.6	2.0
1:1-200 °C-20 h	1.48	0.14	10.8	264.3	88.5	2.9
1:1-250 °C-5 h	1.16	0.13	8.9	371.3	120.7	3.1
1:1-250 °C-10 h	0.77	0.07	11	206.0	47.1	4.9
1:1-250 °C-20 h	0.59	0.05	11.8	252.3	38 .0	6.8
1:1-250 °C-40 h	0.69	0.06	11.5	387.2	76.6	5.0
1:1-300 °C-2 h	0.36	0.035	10.3	606.7	114.3	6.2
1:1-300 °C-5 h[b]	0.33	0.026	13.8	204.8	15.4	11.3

[a] Determined from D = P/S (D measured by $D = l^2/6\theta$), 35 °C, 50 psi.

Supplementary Table 10 O_2 and N_2 diffusion coefficients (D) and solubility coefficients (D) for XPIM-BM-TB at different temperature

Polymer	D [a] (10^{-8} cm^2 s^{-1})			S [a] (10^{-2} cm (STP) cm^{-3} cmHg)		
	O_2	N_2	$(a)_D$	O_2	N_2	$(a)_S$
PIM-BM/TB	1.6	0.38	4.2	264.1	286.8	0.92
1:1-80 °C-20 h	1.4	0.30	4.6	154.1	147.3	1.04
1:1-120 °C-20 h	1.1	0.25	4.4	135.9	130.0	1.05
1:1-200 °C-20 h	0.82	0.17	4.8	107.1	103.5	1.03
1:1-250 °C-10 h	0.55	0.11	5.0	85.1	82.7	1.03
1:1-300 °C-2 h	0.49	0.083	5.9	120.0	113.3	1.06
1:1-300 °C-5 h[b]	0.45	0.071	6.3	40.4	22.5	1.79

[a] Determined from D = P/S (D measured by $D = l^2/6\theta$), 35 °C, 50 psi.

7. Comparison of CO_2 permeabilities and CO_2/CH_4 selectivities

Supplementary Table 11 A comparison of CO_2/CH_4 gas separation data for PIM-1 related membranes

Polymer	P_{CO_2}(Barrer)	P_{CO_2}/P_{CH_4}	T (°C)	Pressure (psi)	Ref.
PIM-EA-TB	7140	10.2	rt	50	17
PIM-1-uv	1364	31.5	rt	60	19
PIM-BM	1689	22.8	35 °C	50	20
PIM-M	4370	17.2	35 °C	50	20
PIM-1	4259	16.5	25 °C	50	20
VinylatedPIM-1 CO50	1370	26	rt	30-100	22
Thioamide-PIM-1	1120	19.6	rt	14.5	23
PIOFG-1	12	117	35	33	24
PIM-BM/TB	2007	20.1	rt	50	This work
PIM-BM/TB	618	21.5	120°C-20h	50	This work
PIM-BM/TB	391	31.7	200 °C-20h	50	This work
PIM-BM/TB	159	543	250 °C-10h	50	This work
PIM-BM/TB	149	79.9	250 °C-20h	50	This work

Supplementary Table 12 A comparison of H_2/CH_4 gas separation data for PIM-1 related membranes

Polymer	P_{H_2}(Barrer)	P_{H_2}/P_{CH_4}	T (°C)	Pressure (psi)	Ref.
PIM-EA-TB	7760	11.1	rt	50	25
PIM-UV 1hr	1488	112.7	rt	50	27
PIM-UV 4hr	452	173.8	rt	50	27
PIM-1	7072	6.5	rt	50	28
PIM/TB (2:8)	1032	22.9	rt	50	28
PIM-1, UV in air, 60 min	1427	33.6	rt	60	19
PIM-1, UV in quar,60 min	1509	50.3	rt	60	19
PIM-PI-3	360	13.3	35 °C	20	29
PIM-PMDA-OH	190	24.7	35 °C	30	30
PIM-6FDA-OH	259	28.5	35 °C	30	30
vinylated PIM-1	1081	4.2	30 °C	--	31
tPBO	4194	28	--	--	32
aPBO	408	35	--	--	32
cPBO	3612	14	--	--	32
sPBO	3585	14	--	--	32
PIM-BM/TB	1925	17.2	rt	50	This work
PIM-BM/TB	839.2	26.9	120°C-20h	50	This work
PIM-BM/TB	720.9	58.5	200 °C-20h	50	This work
PIM-BM/TB	427.1	118.6	250 °C-10h	50	This work
PIM-BM/TB	355.8	197.7	250 °C-20h	50	This work

8. Effect of N_2, O_2 and air atmosphere on PIM-BM, TB and XPIM-BM/TB at 250 °C for 10 h

Supplementary Table 13 Gas transport properties of membranes under N_2, O_2 and air atmosphere at 250 °C-10 h

Polymer	P (Barrer) [a]					Selectivity [b]				
	N_2	CH_4	O_2	H_2	CO_2	CO_2/N_2	CO_2/CH_4	O_2/N_2	H_2/N_2	H_2/CH_4
PIM-BM-70%-0 ppm (O_2/N_2)	110.0	156.0	413.1	1744.1	2131.2	19.4	13.7	3.8	15.9	11.2
PIM-BM-70%-200 ppm (O_2/N_2)	51.1	69.5	183.4	747.7	1228.8	24.0	17.6	3.6	14.6	10.8
PIM-BM -air	2.0	1.4	6.7	27.2	17.3	8.3	12.1	3.3	13.1	19.0
TB-0 ppm (O_2/N_2)	4.2	2.1	22.1	176.1	59.2	14.1	28.2	5.4	44.4	40.0
TB-200 ppm (O_2/N_2)	2.0	1.2	11.1	113.8	36.5	18.1	29.2	5.5	56.3	91.1
TB-air	0.19	0.17	1.3	17.7	2.7	14.4	16.1	6.6	93.3	104.2
PIM-BM/TB-0 ppm (O_2/N_2)	46.4	36.1	178.6	886.6	876.1	18.9	24.2	3.8	19.2	24.6
PIM-BM/TB-50 ppm (O_2/N_2)	37.6	23.9	138.9	747.1	586.3	15.6	24.5	3.7	19.8	31.2
PIM-BM/TB-200 ppm (O_2/N_2)	9.1	3.6	46.8	427.1	197	21.6	54.7	5.3	46.9	118.6
PIM-BM/TB-500 ppm (O_2/N_2)	11.1	6.2	46.4	267.7	218.8	18.7	34.9	4.2	24.0	42.7
PIM-BM/TB-air	0.67	0.95	2.50	54.0	5.82	8.7	6.2	3.7	80.6	57.0

[a] Permeability coefficients measured at 35 °C and 50 psi feed pressure. 1 barrer = 10^{-10} [cm^3 (STP) cm]/(cm^2 s cmHg)

[b] Ideal selectivity $\alpha = (P_a)/(P_b)$

9. Effect of temperature on the XPIM-BM/TB (250 ºC-10 h) membranes

Supplementary Table 14 gas permeation data of XPIM-BM/TB (250 ºC-10 h) at various temperatures

Polymer 250 ºC-10 h	P (Barrer) [a]					Selectivity [b]				
	N_2	CH_4	O_2	H_2	CO_2	CO_2/N_2	CO_2/CH_4	O_2/N_2	H_2/N_2	H_2/CH_4
35 ºC	9.1	3.6	46.8	427.1	197	21.9	54.7	5.3	46.9	118.6
45 ºC	9.9	4.1	53.2	422.3	214.5	21.7	52.3	5.4	42.7	103.0
55 ºC	9.3	4.1	51.8	490.2	216.0	23.2	52.7	5.6	52.7	119.6
0 ºC	0.3	0.2	4.9	46.6	26.9	89.7	134.5	16.3	154.3	233.0
-20 ºC	0.14	0.06	3.6	24.5	10.5	75.0	175.0	25.7	175.0	408.3

[a] Permeability coefficients measured at 35 ºC and 50 psi feed pressure. 1 barrer = 10^{-10} [cm^3 (STP) cm]/(cm^2 s cmHg)

[b] Ideal selectivity $α = (P_a)/(P_b)$

10. Cavity diameters and intensities measured by positron annihilation lifetime spectroscopy

Supplementary Table 15 Cavity diameters and intensities measured by PALS[a]

Polymers	Smaller cavity			Larger cavity		
	Lifetime τ3 (ns)	Cavity diameter (Å)	Intensity I4 (%)	Lifetime τ4 (ns)	Cavity diameter (Å)	Intensity I4 (%)
PIM-BM/TB	1.510±0.148	2.349	5.13±0.34	5.283±0.125	4.870	4.60±0.29
250 ºC-10 h	1.729±0.195	2.586	3.91±0.25	5.345±0.562	4.898	1.94±0.35
300 ºC-5 h	2.023±0.129	2.870	5.45±0.20	6.883±0.886	5.525	1.47±0.27

[a] Measured at 300K

11. Atomic concentration based on XPS

Supplementary Table 16 Atomic concentration of PIM-BM/TB fresh and thermally treated membranes based on XPS

Membrane	Atomic concentration (%)				
	C	O	N	Br	C/O
PIM-BM/TB	74.05	19.33	5.93	0.68	3.83
250 ºC-10 h	73.05	18.90	7.60	0.45	3.86
300 ºC-5 h	72.42	17.52	9.72	0.34	4.13
550 ºC-2 h	85.08	9.49	5.43	--	8.96

12. Membrane performance over 300 °C

Supplementary Table 17 A comparison of CO_2/CH_4 or H_2/CH_4 separation data for thermally treated membranes

Polymer	P_{CO_2}(Barrer)	P_{CO_2}/P_{CH_4}	P_{H_2}(Barrer)	P_{H_2}/P_{CH_4}	T (°C)	Ref.
Crosslinked-PIM-1	2496	26	1666	17	300 °C-12 h	9
Crosslinked-PIM-1	3083	34	2221	25	300 °C-24 h	9
Crosslinked-PIM-1	3339	39	2640	31	300 °C-36 h	9
Crosslinked-PIM-1	4000	55	3872	53	300 °C-48 h	9
6FDA-DAM:DABA	203	24	--	--	300 °C-20 h	35
CA-PIM-1	7672	15.6	7269	14.7	300 °C-2 h	33
6FDA-DAM:DABA	289	28.4	--	--	330 °C-10 h	35
ADHAB-6FDA	27	68	--	--	350 °C-3 h	41
6FDA-mPDA/DABA	2680	52.5	--	--	370 °C-1 h	38
DC-PIM-5	1291	24.6	--	--	375 °C-40 min	21
TOX-PIM-1	5100	17	2979	10	385 °C-1 h	13
TOX-PIM-1	1956	34	2328	40	385 °C-8 h	13
TOX-PIM-1	1680	56	2204	74	385 °C-12 h	13
TOX-PIM-1 (CMS)	1104	69	1820	114	385 °C-24 h	13
CA-PIM-2	9854	5.2	9226	4.9	400 °C-2 h	33
PIM-1-450 (CMS)	28	197	234	1472	450 °C-0.5 h	26
ADHAB-6FDA	151	42	--	--	450 °C-3 h	41
TB–CMS-550	16050	34	14600	31	550 °C-2 h	18
Matrimid CMS 550°C	1250	63	--	--	550 °C-2 h	39
Matrimid CMS 550°C	375	89	--	--	550 °C-8 h	39
6FDA/PMDA–TMMDA	1300	29	--	--	550 °C-2 h	40
6FDA/PMDA–TMMDA	751	36			650 °C-2 h	40
6FDA/BPDA-DAM	4868	54	--	--	550 °C-2 h	36
6FDA/BPDA-DAM	75	72.2			675 °C-2 h	37
6FDA/BPDA-DAM	94	122	--	--	800 °C-0.5 h	36
PIM-6FDA-OH (CMS)	556	93	2177	363	800 °C-0.5 h	34
TB–CMS-800	1406	112	2500	200	800 °C-0.5 h	18
Matrimid CMS 800°C	43.5	200	--	--	800 °C-2 h	39
Torlon	43	18.3	390	166	800 °C-2 h	42
P84	144	67.2	1151	537	800 °C-2 h	42
AD (0.5)-TR-350	43	59	--	--	350 °C-0.5 h	43
AD (0.5)-TR-450	406	37	--	--	450 °C-0.5 h	43
APAF-6FDA TR-350	65	50	--	--	350 °C-0.5 h	43
APAF-6FDA TR-450	974	25	--	--	450 °C-0.5 h	43
TR-PBO	24	71.5	60	184.5	350 °C-2 h	44
TR-PBO	3575	44.2	2856	35.3	450 °C-2 h	44
PBO-MCDEA	67	77.3	35	38.4	400 °C-2 h	45
**PIM-BM/TB**	**68**	**154.5**	**358**	**813.6**	**300 °C-5 h**	**This work**

Supplemental References

(1) X. Chen, Z. Zhang, L. Wu, X. Liu, S. Xu, J. E. Efome, X. Zhang, N. Li, ACS Appl. *Polym. Mater*., 2020, **2**, 987-995.

(2) S. Zhao, J. Liao, D. Li, X. Wang, N. Li, *J. Membr. Sci*., 2018, **566**, 77-86.

(3) B. Yue, Y. Ma, H. Tao, L. Yu, G. Jian, X. Wang, X. Wang, Y. Luc and Z. Hu, *J. Mater. Chem*., 2008, **18**, 1747-1750.

(4) C.Y. Li, W. C. Wang, F.J. Xu, L.Q. Zhang, W.T. Yang, *J. Membr. Sci*., 2011, **367**, 7-13.

(5) E. Papirer, R. Lacroix, J. B. Donnet, G. Nanse, P. Fioux, *Carbon*, 1994, **32**, 1341-1358.

(6) J-F. Colomer, R. Marega, H. Traboulsi, M. Meneghetti, V. Tendeloo, D. Bonifazi, *Chem. Mater*., 2009, **21**, 4747-4749.

(7) R. Gutzler, L. Cardenas, J. Lipton-Duffin, M. E. Garah, Laurentiu E. Dinca, C. E. Szakacs, C. Fu, M. Gallagher, M. Vondrácek, M. Rybachuk, D. F. Perepichk, F. Roseim, *Nanoscale*, 2014, **6**, 2660-2668

(8) A. Basagni, L. Ferrighi, M. Cattelan, L. Nicolas, K. Handrup, L. Vaghi, A. Papagni, F. Sedona, C. D. Valentin, S. Agnoli, M. Sambi, *Chem. Commun*., 2015, **51**, 12593-12596.

(9) F. Li, Y. Xiao, T. Chung, S. Kawi, *Macromolecules*, 2012, **45**, 1427-1437.

(10) K. Halder, P. Georgopanos, S. Shishatskiy, V. Filiz ,V. Abetz, *J. Polym. Sci., Part A: Polym. Chem*., 2018, **56**, 2752-2761.

(11) S. Mukai, *Tetrahedron: Asymmetry*, 1996, **7**, 2671-2674.

(12) F. Ishiwari, N. Takeuchi, T. Sato, Hiroshi Y. Ryota Osuga, J. N. Kondo, T. Fukushima, *ACS Macro Lett*. 2017, **6**, 775-780

(13) Q. Song, S. Cao, R. H. Pritchard1, B. Ghalei, S. A. Al-Muhtaseb, E. M. Terentjev, A. K. Cheetham, E. Sivaniah, *Nature Communications,* 2014, **5**, 4813-4824.

(14) Li, S. H. J. Jo, S. H. Han, C. H. Park, S. Kim, P. M. Budd, Y. M. Lee, *J. Membr. Sci*., 2013, **434**, 137-147.

(15) H. B. Park, C. H. Jung, Y. M. Lee, A. J. Hill, S. J. Pas, S. T. Mudie, E. V. Wagner, B. D. Freeman, D. J. Cookson, *Science*, 2007, **318**, 254-258.

(16) Q. Song, S. Cao, R. H. Pritchard1, B. Ghalei, S. A. Al-Muhtaseb, E. M. Terentjev, A. K. Cheetham, E. Sivaniah, *Nature Communications,* 2014, **5**, 4813-4824

(17) M. Carta, R. Malpass-Evans, M. Croad, Y. Rogan, J. C. Jansen, P. Bernardo, F. Bazzarelli, N. B. McKeown, *Science*, 2013, **339**, 303-307.

(18) Z. Wang, Hg Ren, S. Zhang, F.g Zhang, J. Jin, *ChemSusChem*, 2018, **11**, 916–923

(19). Q. Song, S. Cao, P. Zavala-Rivera, L.P. Lu1, W. Li, Y. Ji, S. A. Al-Muhtaseb, A. K. Cheetham, E. Sivaniah, *Nature Communications*, 2014, **4**, 1918-1926.

(20) S. Zhao, J. Liao, D. Li, X. Wang, N. Li, *J. Membr. Sci*., 2018, **566**, 77-86.

(21) N. Du, M. M. Dal-Cin, G. P. Robertson, M. l. D. Guiver, *Macromolecules*, 2012, **45**, 5134-5139.

(22) K. Halder, S. Neumann, G.a Bengtson, M. Munir Khan, V. Filiz, V. Abetz, *Macromolecules*, 2018, **51**, 7309-7319.

(23) C. R. Mason, L. Maynard-Atem, K. W. J. Heard, B. Satilmis, P. M. Budd, K. Friess, M. Lanč, P. Bernardo, G. Clarizia, J. C. Jansen, *Macromolecules*, 2014, **47**, 1021-1029.

(24) H. B. Park, C. H. Jung, Y. M. Lee, A. J. Hill, S. J. Pas, S. T. Mudie, E. V. Wagner, B. D. Freeman, D. J. Cookson, *Science*, 2007, **318**, 254-258.

(25) M. Carta, R. Malpass-Evans, M. Croad, Y. Rogan, J. C. Jansen, P. Bernardo, F. Bazzarelli, N. B. McKeown, *Science*, 2013, **339**, 303-307.

(26) S. He, X. Jiang, S. Li, F. Ran, J. Long, L. Shao, *AIChE J*,. 2020, **66**, e16543.

(27) F. Y. Li, Y. Xiao, Y. K, Ong, T.S. Chung, *Adv Energy Mater*., 2012, **2**, 1456-1466.

(28) S. Zhao, J. Liao, D. Li, X. Wang, N. Li, *J. Membr. Sci*., 2018, **566**, 77-86.

(29). B. S. Ghanem, N. B. McKeown, P. M. Budd, J. D. Selbie, D. Fritsch, *Adv. Mater*., 2008, **20**, 2766−2771.

(30) Xiaohua. Ma, R. S. Youssef Belmabkhout, Y. Zhu, E. Litwiller, M. Jouiad, I. Pinnau, Y. Han, *Macromolecules*, 2012, **459**, 3841-3849.

(31) K. Halder, S. Neumann, G.a Bengtson, M. Munir Khan, V. Filiz, V. Abetz, *Macromolecules*, 2018, **51**, 7309-7319.

(32) S. H. Han, N. Misdan, S. Kim, C. M. Doherty, A. J. Hill, Y. M. Lee, *Macromolecules*, 2010, **43**, 7657-7667.

(33) T. Zhang, L. Deng, P. Li, *Ind. Eng. Chem. Res*., 2020, ***59***, 18640-18648.

(34) X. Ma, R. Swaidan, B. Teng, H. Tan, O. Salinas, E. Litwiller, Y. Han, I. Pinnau, *Carbon*, 2013, **62**, 88-96.

(35) W. Qiu, C. Chen, L. Xu, L. Cui, D. R. Paul, W. J. Koros, *Macromolecules*, 2011, **44**, 6046–6056.

(36) M. Kiyono, P. J. Williams, W. J. Koros, *J. Membr. Sci,* 2010, **359**, 2-10.

(37) Y. Cao, K. Zhang, O. Sanyal, W. J. Koros, *Angew. Chem. Int. Ed*. 2019, **58**, 1–6.

(38) W. Qiu, K. Zhang, F. S. Li, K. Zhang, W. J. Koros, *ChemSusChem*, 2014, **7**, 1186–1194.

(39) D. Q. Vu, W. J. Koros, S. J. Miller, *J. Membr. Sci,* 2003, **21**1, 311-334.

(40) L. Shao, T. Chung, G. Wensley, S. H. Goh, K. P. Pramoda, *J. Membr. Sci*, 2004, **244**, 77-87.

(41) C. Aguilar-Lugo, C. Á lvarez, Y. M. Lee, J. Campa, Á. E. Lozano, *Macromolecules*, 2018, **51**, 1605−1619.

(42) S. S. Hosseini, T. S. Chung, *J. Membr. Sci*., 2009, **328**, 174–185.

(43) C. Aguilar-Lugo, C. Álvarez, Y. M. Lee, J. G. de la Campa, Á. E. Lozano, *Macromolecules*, 2018, **51**, 1605−1619.

(44) S. Kim, H. Jin Jo, Y. M. Lee, *J. Membr. Sci*, 2013, **441**, 1-8.

(45) Y. Zhuang, J. G. Seong, W. H. Lee, Y. S. Do, M. J. Lee, G. Wang, M. D. Guiver, Y .M. Lee, *Macromolecules*, 2015, **48**, 5286−5299.
